# Integrated analysis and identification of hub genes as novel biomarkers for Alzheimer’s disease

**DOI:** 10.3389/fnagi.2022.901972

**Published:** 2022-08-30

**Authors:** Kun Zhao, Hui Zhang, Yinyan Wu, Jianzhi Liu, Xuezhong Li, Jianyang Lin

**Affiliations:** ^1^Department of Neurology, Affiliated People’s Hospital of Jiangsu University, Zhenjiang, China; ^2^Fujian Center for Safety Evaluation of New Drug, Fujian Medical University, Fuzhou, China; ^3^Department of General Surgery, Affiliated People’s Hospital of Jiangsu University, Zhenjiang, China

**Keywords:** Alzheimer’s disease (AD), novel biomarker, integrated analysis, immune, hub gene, AGAP3

## Abstract

Alzheimer’s disease (AD) is an intractable and progressive neurodegenerative disorder that can lead to severe cognitive decline, impaired speech, short-term memory loss, and finally an inability to function in daily life. For patients, their families, and even all of society, AD can impart great emotional pressure and economic costs. Therefore, this study aimed to investigate potential diagnostic biomarkers of AD. Using the Gene Expression Omnibus (GEO) database, the expression profiles of genes were extracted from the GSE5281, GSE28146, and GSE48350 microarray datasets. Then, immune-related genes were identified by the intersections of differentially expressed genes (DEGs). Functional enrichment analyses, including Gene Ontology, Kyoto Encyclopedia of Genes and Genomes, Disease Ontology (DO), and Gene Set Enrichment Analysis (GSEA), were performed. Subsequently, random forest models and least absolute shrinkage and selection operator regression were used to further screen hub genes, which were then validated using receiver operating characteristic (ROC) curve analysis. Finally, 153 total immune-related DEGs were identified in relation to AD. DO analysis of these immune-related DEGs showed that they were enriched in “lung disease,” “reproductive system disease,” and “atherosclerosis.” Single GSEA of hub genes showed that they were particularly enriched in “oxidative phosphorylation.” ROC analysis of *AGAP3* yielded an area under the ROC curve of 0.878 for GSE5281, 0.727 for GSE28146, and 0.635 for GSE48350. Moreover, immune infiltration analysis demonstrated that AGAP3 was related to follicular helper T cells, naïve CD4 T cells, naïve B cells, memory B cells, macrophages M0, macrophages M1, macrophages M2, resting natural killer (NK) cells, activated NK cells, monocytes, neutrophils, eosinophils, and activated mast cells. These results indicate that identifying immune-related DEGs might enhance the current understanding of the development and prognosis of AD. Furthermore, *AGAP3* not only plays a vital role in AD progression and diagnosis but could also serve as a valuable target for further research on AD.

## Introduction

Alzheimer’s disease (AD) is the leading cause of neurodegeneration and dementia; clinically, it manifests as behavioral dysfunction, memory loss, and cognitive impairment (Joe and Ringman, 2019; Li et al., 2021). More than 47 million people are estimated to have dementia, and among them, AD cases account for 60–80% (Anand et al., 2017). The pathological features of AD are synaptic dysfunction, tau-containing neurofibrillary tangles, amyloid plaques [mainly amyloid-β (Aβ)], and neuronal loss (Knopman et al., 2021). However, to date no effective biomarkers or mechanisms have been identified for the prediction, diagnosis, or treatment of AD.

Many strategies, such as stimulating cognition, exercise, and lifestyle changes, can prevent AD and help improve or maintain cognitive functions in elderly people, who are at a high risk of AD (Ngandu et al., 2015). Unfortunately, to date, no effective treatment for AD has been developed. Currently, patients with AD are treated through pharmacotherapy, such as N-methyl-D-aspartate (NMDA) receptor antagonists, memantine, and cholinesterase inhibitors. Patients with AD often suffer from comorbidities. (Patients with complications can undergo pharmacological interventions, including lower doses or more suitable instruments), but there is currently no comprehensive therapeutic intervention for AD. Immune cells at different stages of AD can lead to serious dynamic damage and might have many heterogeneous functions (Jevtic et al., 2017). Innate immune cells in peripheral blood, including neutrophils, monocytes, or natural killer (NK) cells, can be recruited into the central nervous system (CNS) to participate in the development of AD (Prinz and Priller, 2017; Castellani and Schwartz, 2020; Wu et al., 2021). Therefore, to improve the diagnosis and treatment of AD while enhancing the patients’ immune system, there is an urgent need to explore and identify novel biomarkers and therapeutic targets related to immune cells. This can be achieved by screening genes and gene-networks for changes related to AD onset and development.

The metabolism of immune cells also changes in patients with AD; this change could be related to disease pathology (Butterfield and Halliwell, 2019). Furthermore, autoimmunity has been shown to have an effect on these patients (Lim et al., 2021). In this study, therefore, AD datasets collected from the Gene Expression Omnibus (GEO) database were used to conduct systemic analyses (Barrett et al., 2013). First, transcript-level differential analysis was conducted to obtain differentially expressed genes (DEGs). Second, hub genes were identified using the least absolute shrinkage and selection operator (LASSO) and random forest analysis to more rigorously screen biomarkers in a standardized manner. Correlations between identified theoretical biomarker(s) and immune infiltrating cells were then analyzed with CIBERSORT to reveal their diagnostic value regarding AD.

## Materials and methods

### Datasets

The primary data of three gene expression profile datasets, GSE5281 (Liang et al., 2008a,b; Readhead et al., 2018), GSE28146 (Blalock et al., 2011), and GSE48350 (Berchtold et al., 2008; Blair et al., 2013), were downloaded from the National Center for Biotechnology Information (NCBI) GEO database^1^ (Barrett et al., 2013). GSE5281 contains data from 87 AD tissues and 74 normal tissues, GSE28146 contains data from 22 AD tissues and eight normal tissues, and GSE48350 contains data from 80 AD tissues and 173 normal tissues. The clinical data and the tissue collection methods of the three datasets are showed in Supplementary Tables 1, 2. In addition, the tissues of two datasets (GSE5281, GSE28146), used for sequencing, are all in terms of laser capture microdissection from Frozen and fixed samples; the tissues of GSE48350, used for sequencing, are in terms of extracting RNA from Frozen unfixed samples. All the gene expression profile datasets were derived from the GPL570 platform (HG-U-133_Plus_2) Affymetrix Human Genome U133 Plus 2.0 Array. Moreover, hallmark gene sets were downloaded from the MsigDB database^2^ (Subramanian et al., 2005; Liberzon et al., 2015), whereas the immune-related genes of humans were downloaded from the Immport database^3^ (Bhattacharya et al., 2018).

### Differentially expressed genes

To analyze the DEGs between the AD and normal groups, the DealGPL570 package was used to process the primary data of the three datasets (GSE5281, GSE28146, and GSE48350). DEGs were screened by setting the fold-change (FC) threshold to | log FC| > 0.5 and adjusting the significance to *P* < 0.05 using the R package “limma” (Ritchie et al., 2015; Phipson et al., 2016). Each gene (e.g., g) was assigned vector of gene expression values (yg) and a design matrix X that related these values to some coefficients of interest (βg). The limma package includes statistical methods that facilitate information borrowing using empirical Bayes methods to obtain posterior variance estimators (sg2*), incorporate observation weights (wgj, where j refers to the sample) to allow for variations in data quality, and facilitate variance modeling to accommodate technical or biological heterogeneity that could be present, as well as pre-processing methods such as variance stabilization to reduce noise (Ritchie et al., 2015). We used Benjamin and Hochberg’s method (BH) to control the false discovery rate (Hochberg, 1995). The statistical methods we used were moderate t-statistics, ordinary t-statistics, empirical Bayesian, the BH method, and B- and F-statistics (Hochberg, 1995; Smyth, 2004; Phipson et al., 2016). The DEGs were then visualized using a heatmap. Next, immune-related genes were intersected with the DEGs of each of the three datasets separately, thereby obtaining immune-related DEGs; these are shown using a volcano plot.

### Construction of protein–protein interaction networks of immune differentially expressed genes

The immune DEGs of each of the three datasets were utilized to build three co-expression networks using the STRING database^4^ (Szklarczyk et al., 2019). Cytoscape v3.8.2 (Shannon et al., 2003) was then used to construct all three protein–protein interaction (PPI) networks, and the NetworkAnalyst plug-in was used to calculate the node degrees and to demonstrate the PPI networks. The immune DEGs of the three databases were then intersected to identify the hub genes. Furthermore, a competing endogenous ribonucleic acid (ceRNA) network [messenger RNA (mRNA)–micro RNA (miRNA)–long non-coding RNA (lncRNA)] was constructed for every hub gene using starBase^5^ (Li et al., 2014), miRDB^6^ (Chen and Wang, 2020), and miRWalk^7^ (Sticht et al., 2018).

### Enrichment analysis

As few hub genes were identified, all immune DEGs of the three datasets were combined to conduct Gene Ontology [GO, including molecular function (MF), biological process (BP), and cellular component (CC)], Kyoto Encyclopedia of Genes and Genomes (KEGG) and Disease Ontology (DO) enrichment analyses. Furthermore, single-gene Gene set enrichment analysis (GSEA) was conducted for each hub gene. The normalized enrichment score and the nominal *P*-value were used to calculate and sort the enriched pathways for each hub gene. C2.all.v6.2.symbols.gmt was used as the reference gene set, and 1,000 permutations of the gene set were created. The clusterProfilter package was used to perform all enrichment analyses.

### Immune infiltration

CIBERSORT, which quantifies cell fractions based on tissue gene expression profiles, is a universal computational method. CIBERSORT is an analytical tool from the Alizadeh Lab developed by Newman et al. (2015, 2019) to provide an estimation of the abundances of member cell types in a mixed cell population, using gene expression data. Through the use of the linear support vector regression, CIBERSORT could estimate the abundance of immune cells through deconvolution of the expression matrix of immune cell sub types. In addition, CIBERSORT provides 22 frequent immune infiltrating cells, including immune cells of different cell types and cell functions (Chen et al., 2018). Immune infiltration was assessed based on the three datasets using the CIBERSORT algorithm to identify differences in immune cell infiltration between the AD and normal groups. The R package “ggplot2” was used to visualize the data through bar charts, correlation heat maps, heat maps, and violin plots.

### Identification and validation of diagnostic biomarkers

To identify diagnostic biomarkers related to AD, random forest models and LASSO regression analysis were used to filter the GSE5281 dataset. The R package “glmnet” (Friedman et al., 2010) was used to implement the LASSO regression analysis, and the R package “Random Forest” (Wiener, 2002) was used to implement the random forest models. Glmnet is a package that fits generalized linear and similar models via penalized maximum likelihood. The stability of the LASSO models was assessed by creating 1,000 models using the same training set but different seeds during the 10-fold cross-validation for the optimum lambda. In addition, random forest models were constructed based on the full data set. The number of trees was selected to minimize the out-of-bag error rate, and the number of random variables used in each tree was optimized using the tuning function (tuneRF, randomForest; Liaw and Wiener, 2001). Then, the intersections of the two algorithms were taken as the final result. Receiver operating characteristic (ROC) curve analysis of the GSE5281 dataset was conducted to verify the result of the LASSO and random forest models. Then, ROC curve analysis was conducted to verify their accuracy in the GSE28146 and GSE48350 datasets. Finally, those genes for which the area under the curve (AUC) was > 0.6 were selected to analyze their correlations with immune infiltrating cells. The expression of diagnostic biomarkers was divided into two groups (high and low) according to median values. Finally, the differences in immune infiltrating cells between high expressed and low expressed diagnostic biomarkers were plotted as boxplots to allow for comparisons.

### Statistical analysis

All experiments were conducted using the R software (version: 4.1.1).^8^ We used the BH method to perform multiple test correction, which was performed to reduce false positive rates in multiple tests. The Student’s *t*-test was applied to assess the statistical significance of normally distributed variables, and the Mann-Whitney *U*-test (i.e., Wilcoxon rank-sum test) was applied to estimate the independence of and differences among non-normally distributed variables. Spearman coefficients were used for the correlation between genes and immune cells. All statistical tests were two-tailed, and a *P*-value < 0.05 was considered statistically significant.

## Results

### Immune-related differentially expressed genes

**FIGURE 1:**
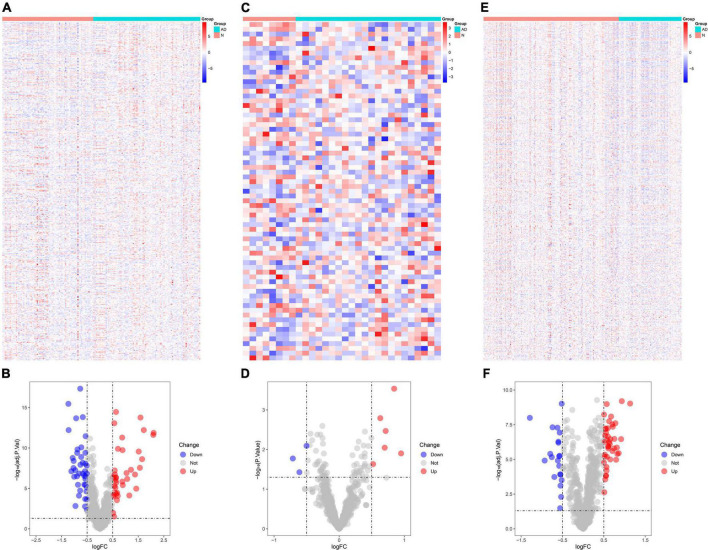
Identification of differentially expressed genes (DEGs) in Alzheimer’s disease tissues and normal tissues. **(A)** Heat map demonstrates the DEGs in GSE5281. **(B)** Volcano plot demonstrates the immune DEGs in GSE5281. **(C)** Heat map demonstrates the DEGs in GSE28146. **(D)** Volcano plot demonstrates the immune DEGs in GSE28146. **(E)** Heat map demonstrates the DEGs in GSE48350. **(F)** Volcano plot demonstrates the immune DEGs in GSE48350. *P < 0.05. * The difference is significant.

The number of DEGs identified in GSE5281 was 800 (Figure 1A), of which 80 were immune-related DEGs (Figure 1B). The number of DEGs identified in GSE28146 was 200 (Figure 1C), of which nine were immune-related DEGs (Figure 1D). The number of DEGs identified in GSE48350 was 640 (Figure 1E), including 64 immune-related DEGs (Figure 1F).

### Construction of protein-protein interaction and competing endogenous ribonucleic acid networks

**FIGURE 2:**
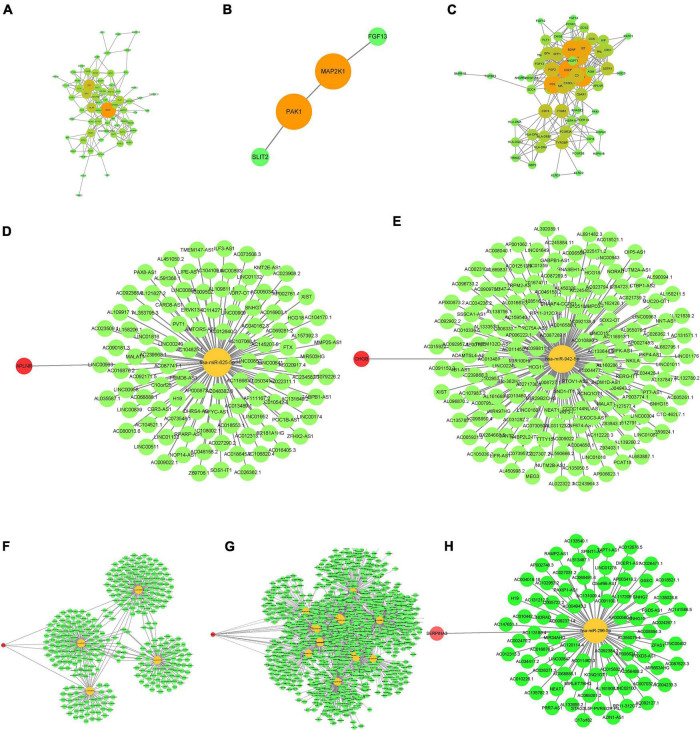
Construction of PPI (protein-protein interaction) and ceRNA (mRNA-miRNA-lncRNA) networks of the immune DEGs. **(A–C)** PPI network. **(A)** GSE5281. **(B)** GSE28146. **(C)** GSE48350. **(D–H)** ceRNA network. **(D)** APLNR. **(E)** CHGB. **(F)** FGF13. **(G)** PAK1. **(H)** SERPINA3.

The PPI networks for the three datasets are shown in Figures 2A–C; the darker the color of the nodes, the higher the confidence and the stronger the co-expression. The intersections of the immune-related DEGs of the three databases resulted in the identification of the following hub genes: *APLNR*, *CHGB*, *FGF13*, *PAK1*, and *SERPINA3*. CeRNA networks for each hub gene are shown in Figures 2D–H, in which yellow nodes are hub genes, green nodes are miRNA, and red nodes are lncRNA.

### Enrichment analysis

**FIGURE 3:**
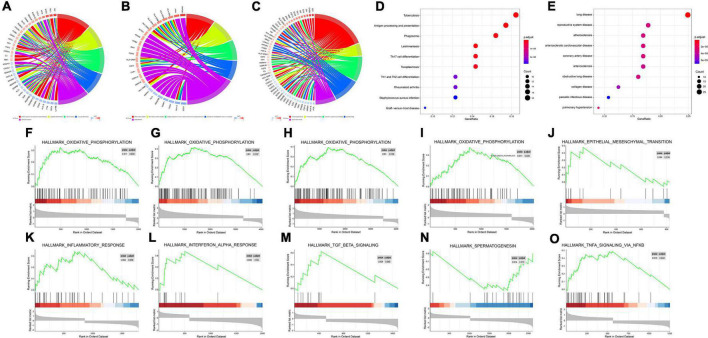
Enrichment analyses (GO, KEGG, DO, GSEA) of immune DEGs. **(A)** Circos diagram depicting GO-BP Enrichment analysis. **(B)** Circos diagram depicting GO-CC Enrichment analysis. **(C)** Circos diagram depicting GO-MF Enrichment analysis. **(D)** Dot plot depicting KEGG Enrichment analysis. **(E)** Dot plot depicting DO Enrichment analysis. **(F–O)** Signal-gene gene set enrichment analysis (GSEA) indicating statistically significant enrichment from AD and normal tissues, and representative hallmarks. **(F)** APLNR in GSE5281. **(G)** CHGB in GSE5281. **(H)** FGF13 in GSE5281. **(I)** PAK1 in GSE5281. **(J)** CHGB in GSE28146. **(K)** APLNR in GSE48350. **(L)** CHGB in GSE48350. **(M)** FGF13 in GSE48350. **(N)** PAK1 in GSE48350. **(O)** SERPINA3 in GSE48350.

GO enrichment analysis of the immune DEGs of the three datasets combined showed that they were significantly enriched in “active regulation of response to external stimuli,” “regulation of chemotaxis,” and “cell chemotaxis” (Figures 3A–C). KEGG enrichment analysis, meanwhile, demonstrated that these genes were significantly enriched in “tuberculosis,” “phagosome,” and other pathways (Figure 3D). DO enrichment analysis indicated that these genes were significantly enriched in “reproductive system disease,” “pulmonary disease,” “arteriosclerosis,” and other disease pathways (Figure 3E).

Meanwhile, for every immune-related DEG (*n* = 5), divided into high or low groups based on expression, single-gene GSEA was performed separately on each dataset. Single-gene GSEA demonstrated that these genes were significantly enriched in the “HALLMARK_OXIDATIVE_PHOSPHORYLATION,” “HALL- MARK_OXIDATIVE_PHOSPHORYLATION,” “HALL- MARK_OXIDATIVE_PHOSPHORYLATION,” “HALLMARK_OXIDATIVE_PHOSPHORYLATION,” “HALLMARK_EPITHELIAL_MESENCHYMAL_ TRANSITION,” “HALLMARK_INFLAMMATORY_ RESPONSE,” “HALLMARK_INTERFERON_ALPHA_ RESPONSE,” “HALLMARK_TGF_BETA_SIGNALING,” “HALLMARK_ SPERMATOGENESI,” “HALLMARK_TNFA_ SIGNALING_VIA_NFKB,” and other pathways (Figures 3F–O). Table 1 shows the detailed results.

**TABLE 1:** GO, KEGG, DO, and GSEA enrichment analysis.

ID	Description	Count in gene set	p.adjust
**GO-BP**			
GO:0032103	Positive regulation of response to external stimulus	23	4.55E-14
GO:0050920	Regulation of chemotaxis	16	1.29E-09
GO:0060326	Cell chemotaxis	18	1.29E-09
GO:0030595	Leukocyte chemotaxis	16	1.29E-09
GO:0050900	Leukocyte migration	21	7.62E-09
GO:0051047	Positive regulation of secretion	19	2.69E-08
GO:1903532	Positive regulation of secretion by cell	18	6.01E-08
GO:0001667	Ameboidal-type cell migration	19	7.08E-08
GO:0001819	Positive regulation of cytokine production	19	7.08E-08
GO:0032103	Positive regulation of response to external stimulus	19	4.55E-14
**GO-CC**			
GO:0042613	MHC class II protein complex	8	2.48E-12
GO:0071556	Integral component of lumenal side of endoplasmic reticulum membrane	7	2.38E-08
GO:0098553	Lumenal side of endoplasmic reticulum membrane	7	2.38E-08
GO:0005925	Focal adhesion	16	1.92E-07
GO:0005924	Cell-substrate adherens junction	16	1.92E-07
GO:0030055	Cell-substrate junction	16	1.92E-07
GO:0009897	External side of plasma membrane	15	6.54E-07
GO:0012507	ER to Golgi transport vesicle membrane	7	2.81E-06
GO:0101002	Ficolin-1-rich granule	10	5.50E-06
**GO-MF**			
GO:0023023	MHC protein complex binding	7	4.30E-08
GO:0001664	G protein-coupled receptor binding	14	8.37E-07
GO:0042277	Peptide binding	14	9.94E-07
GO:0008083	Growth factor activity	11	9.94E-07
GO:0019955	Cytokine binding	10	9.94E-07
GO:0019838	Growth factor binding	10	1.64E-06
GO:0023026	MHC class II protein complex binding	5	2.39E-06
GO:0033218	Amide binding	14	5.22E-06
GO:0005179	Hormone activity	9	5.22E-06
**KEGG**			
hsa04612	Antigen processing and presentation	14	4.42E-15
hsa05140	Leishmaniasis	18	2.63E-11
hsa05152	Tuberculosis	14	2.31E-10
hsa04659	Th17 cell differentiation	16	1.14E-09
hsa04145	Phagosome	14	1.14E-09
hsa05145	Toxoplasmosis	12	1.78E-09
hsa04658	Th1 and Th2 cell differentiation	12	2.41E-08
hsa05323	Rheumatoid arthritis	9	2.41E-08
hsa05332	Graft-versus-host disease	12	2.80E-08
hsa05150	Staphylococcus aureus infection	17	2.80E-08
**DO**			
DOID:850	Lung disease	10	9.12E-08
DOID:6432	Pulmonary hypertension	18	3.07E-05
DOID:2320	Obstructive lung disease	19	3.07E-05
DOID:1936	Atherosclerosis	19	3.07E-05
DOID:2348	Arteriosclerotic cardiovascular disease	19	3.07E-05
DOID:3393	Coronary artery disease	20	3.07E-05
DOID:15	Reproductive system disease	19	3.07E-05
DOID:2349	Arteriosclerosis	14	3.49E-05
DOID:854	Collagen disease	12	5.18E-05
DOID:1398	Parasitic infectious disease	28	7.05E-05

**MSigDB collection GSEA**	**Gene set name**	**NOM *p*-val**	**FDR *q*-val**

h.all.v6.1.symbols.gmt	HALLMARK_OXIDATIVE_PHOSPHORYLATION	0.001	0.023
	HALLMARK_OXIDATIVE_PHOSPHORYLATION	0.001	0.019
	HALLMARK_OXIDATIVE_PHOSPHORYLATION	0.001	0.019
	HALLMARK_OXIDATIVE_PHOSPHORYLATION	0.001	0.028
	HALLMARK_EPITHELIAL_MESENCHYMAL_TRANSITION	0.016	0.213
	HALLMARK_INFLAMMATORY_RESPONSE	0.002	0.010
	HALLMARK_INTERFERON_ALPHA_RESPONSE	0.003	0.052
	HALLMARK_TGF_BETA_SIGNALING	0.003	0.027
	HALLMARK_SPERMATOGENESI	0.001	0.020
	HALLMARK_TNFA_SIGNALING_VIA_NFKB	0.002	0.004

### Screening of diagnostic markers

**FIGURE 4:**
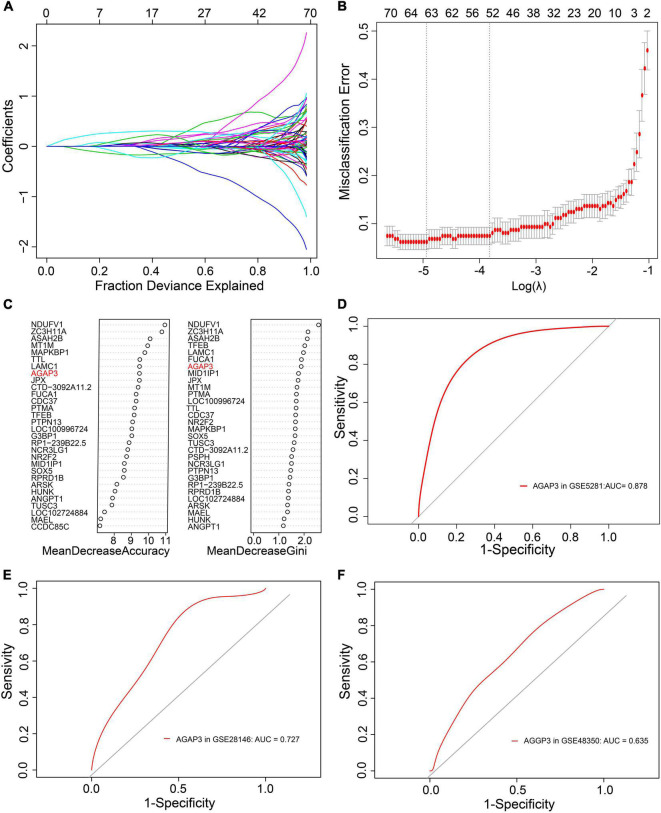
AGAP3 is the diagnostic marker of AD patients. **(A,B)** LASSO regression analysis model. **(C)** Random Forest models; MeanDecreaseAccuracy and MeanDecreaseGini. **(D)** The ROC curve of AGAP3 in GSE5281. **(E)** The ROC curve of AGAP3 in GSE28146. **(F)** The ROC curve of AGAP3 in GSE48350.

During the screening of diagnostic markers, 63 were filtered by LASSO regression analysis (Figures 4A,B), whereas 29 were filtered through random forest models (Figure 4C). An intersection of the two methods identified 10 genes. ROC analysis of these genes were showed in Table 2 and Supplementary Figure 1, and revealed that only the AUC of *AGAP3* > 0.6. Respectively, *AGAP3* exhibited an AUC of 0.878 in GSE5281 (Figure 4D), 0.727 in GSE28146 (Figure 4E), and 0.635 in GSE48350 (Figure 4F). Thus, *AGAP3* was selected as the final diagnostic marker.

**TABLE 2:** The results of ROC analysis.

Data set	ACIN1	BC040734	HINT3	LINC00936	PTMA	RAB30	LOC100996724	LOC102724884	LOC102724927	AGAP3
GSE5281	0.67	0.766	0.678	0.691	0.863	0.525	0.857	0.743	0.751	0.878
GSE28146	0.596	0.515	0.529	0.548	0.497	0.472	0.576	0.599	0.637	0.727
GSE48350	0.601	0.648	0.588	0.724	0.654	0.51	0.573	0.614	0.599	0.635

#### Immune infiltration

**FIGURE 5:**
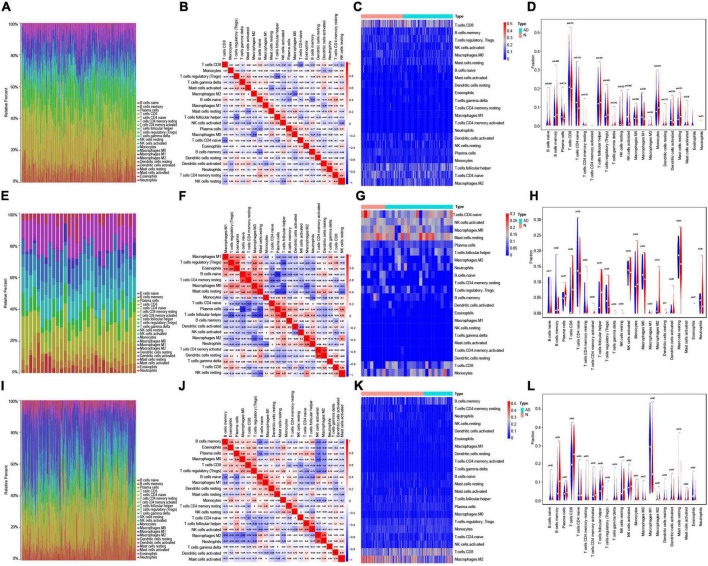
Immune infiltration analysis in AD. **(A)** Bar plot showing the proportion of 22 types immune infiltrating cells in GSE5281. **(B)** Correlation heatmap showing the correlation between immune infiltrating cells in GSE5281. **(C,D**) Heatmap **(C)** and violin plot **(D)** showing the expression difference of immune infiltrating cells between AD and normal samples in GSE5281. **(E)** Bar plot showing the components of 22 types immune infiltrating cells in GSE28146. **(F)** Correlation heatmap showing the correlation between immune infiltrating cells in GSE28146. **(G,H)** Heatmap **(G)** and violin plot **(H)** showing the expression difference of immune infiltrating cells between AD and normal samples in GSE28146. **(I)** Bar plot showing the components of 22 types immune infiltrating cells in GSE48350. **(J)** Correlation heatmap showing the correlation between immune infiltrating cells in GSE48350. **(K,L)** Heatmap **(K)** and violin plot **(L)** showing the expression difference of immune infiltrating cells between AD and normal samples in GSE48350.

Based on each dataset, the proportions of the 22 types of immune infiltrating cells that were analyzed are shown in bar plots (Figures 5A,E,I). The correlations between immune infiltrating cells are visualized by a correlation heatmap (Figures 5B,F,J). The differences in immune infiltrating cells between AD and normal samples are shown as heat maps and violin plots for GSE5281 (Figures 5C,D), GSE28146 (Figures 5G,H), and GSE48350 (Figures 5K,L). Consistent with the immune infiltration results of the three datasets, resting CD4 memory T cells, CD4 naïve T cells, naïve B cells, memory B cells, plasma cells, resting NK cells, activated NK cells, monocytes, macrophages M0, macrophages M1, macrophages M2, activated mast cells, eosinophils, and neutrophils were significantly different between AD and normal samples.

### Correlations between diagnostic gene expression and infiltrating levels of immune cells in Alzheimer’s disease

**FIGURE 6:**
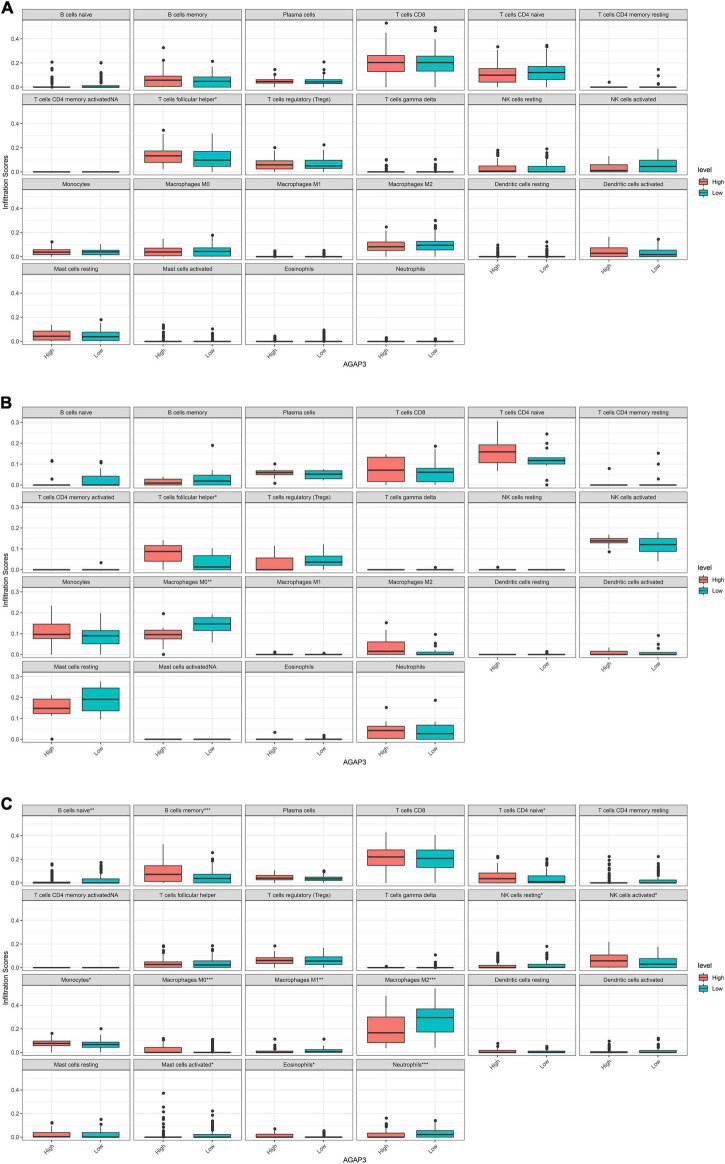
Correlation between diagnostic gene expression and infiltration levels of immune cells in AD. **(A)** The correlation of between AGAP3 and immune infiltrating cells in GSE5281. **(B)** The correlation of between AGAP3 and immune infiltrating cells in GSE28146. **(C)** The correlation of between AGAP3 and immune infiltrating cells in GSE48350. *P < 0.05, **P < 0.01, ***P < 0.001.

*AGAP3*, exhibited correlations with immune infiltrating cells in each dataset. Figure 6 demonstrates that differences in 22 types of immune cells were identified between the high and low *AGAP3* groups. Follicular helper T cells, macrophages M0, naïve B cells, memory B cells, naïve CD4 T cells, resting NK cells, activated NK cells, monocytes, macrophages M0, macrophages M1, macrophages M2, activated mast cells, eosinophils, and neutrophils all exhibited significant differences.

## Discussion

AD and relevant dementias are challenging conditions that seriously affect patients and their families (Ren et al., 2022). Furthermore, there are currently no good methods or drugs to help patients with AD to recover or gain an improved quality of life, and the exact mechanism of AD remains elusive. From a data perspective, recent studies have focused on exploring effective targets for AD (Chong et al., 2021); some genes, including oncogenes and suppressor genes, have been found, such as β-secretase 1 (*BACE1*) (Dai et al., 2021) and receptor-interacting protein kinase 1 (*RIPK1*) (Li et al., 2021). Nevertheless, novel biomarkers with high sensitivity, specificity, and efficiency are necessary to improve AD diagnosis, understand immune infiltration, and treat this disease. Thus, it is important to develop predictive models that can assess prospective biomarkers. In the present study, based on bioinformatics analysis, the development gene expression in AD were investigated through the systematic analyses of three expression profiles from the GEO database. Immune-related DEGs and the prognostic values of hub genes were then investigated.

In these three expression profiles, which included data from a total of 189 AD tissues and 255 normal tissues, 153 immune-related DEGs were screened for further analyses. A PPI network was constructed for these immune-related DEGs, and five hub genes (*CHGB*, *APLNR*, *FGF13*, *PAK1*, and *SERPINA3*) were identified as promising prognostic and diagnostic targets for AD. CeRNA networks were therefore built (mRNA-miRNA-lncRNA) for every hub gene. Functional and pathway enrichment analyses revealed that the immune-related DEGs were enriched in biological processes including the positive regulation of responses to external stimuli; regulation of chemotaxis; cell chemotaxis cellular component major histocompatibility complex (MHC) protein complex; MHC class II protein complex, an integral component of the luminal side of endoplasmic reticulum membrane molecular function receptor ligand activity; MHC protein complex binding; and G protein-coupled receptor binding. Thus, immune cell infiltration appears to be closely related to AD. Moreover, the identified immune-related DEGs could be closely related to lung disease, reproductive system disease, and atherosclerosis. In addition, in accordance with the results of single-gene GSEA, oxidative phosphorylation was found to be enriched based on four hub genes (*CHGB*, *APLNR*, *FGF13*, and *PAK1*). LASSO regression analysis and random forest models were used to identify biomarkers related to AD. ROC curve verification revealed that only *AGAP3* demonstrated high predictive performance in terms of specificity and sensitivity.

ArfGAP with a GTPase domain, ankyrin repeat, and PH domain 3 (*AGAP3*), which encodes an essential component of the NMDA receptor signaling complex, mediates long-term potentiation in synapses by linking the activation of NMDA receptors to alpha-amino-3-hydroxy-5-methyl-4-isoxazolepropionic acid (AMPA) receptor trafficking (Oku and Huganir, 2013). *AGAP3* is a guanosine triphosphate containing a nuclear localization signal sequence, first identified in 2006 (Qin et al., 2006). Qin revealed that *AGAP3* is closely related to the generation of reactive oxygen species and to the ubiquitin–proteasome pathway (Qin et al., 2006). *AGAP3* can protect cells and mediate the functions of the antioxidant pathway by restraining the accumulation of unfolded proteins (Nagashima et al., 2011). Furthermore, as it contains multiple domains (a pleckstrin homology domain, GTPase-like domain, and ArfGAP domain), *AGAP3* could be a component of the NMDA receptor complex, which links NMDA receptor activation to the regulation of alpha-amino-3-hydroxy-5-methyl-4-isoxazolepropionic acid (AMPA) trafficking and the plasticity of synapses (Oku and Huganir, 2013). In addition, *AGAP3* has been shown to be most highly expressed in the brain in the Human Protein Atlas (HPA) database, through analyses of human tissue-specific expression (Fagerberg et al., 2014). Moreover, *AGAP3* mainly participates in the endocytosis pathway (Kanehisa and Goto, 2000). Given their importance with respect to many receptors associated with human diseases (such as AMPA and NMDA), ArfGAPs could represent a novel therapeutic target, in addition to providing mechanistic insights into receptor sorting (Shiba and Randazzo, 2014). To date, however, there has been no related research on the development or underlying mechanism of AD pathogenesis. In this study, the potential prognostic and diagnostic involvement of different markers in AD was comprehensively analyzed, demonstrating that *AGAP3* had AUCs of 0.727 and 0.635 in the GSE28146 and GSE48350 datasets, respectively for AD. Thus, *AGAP3* is a good diagnostic and prognostic biomarker with favorable sensitivity and specificity. It represents a promising diagnostic target for AD development; this may aid in the early detection of AD and improved treatment options for patients with AD.

Owing to the blood brain barrier, the brain is traditionally considered immune-privileged; peripheral immune cells are rarely detected in the brain parenchyma (Jevtic et al., 2017). However, in AD, clinical and experimental studies have demonstrated that peripheral immune cells (macrophages, monocytes, and neutrophils) participate in inflammatory responses and Aβ metabolism (Ennerfelt and Lukens, 2020). CD4^+^ cells, numbers of which are positively correlated with AD, have been revealed to be a hub factor of cognitive dysfunction in AD (Pérez-González et al., 2021), as have CD8^+^ cells (Unger et al., 2020). Immunoglobulin accumulation around Aβ plaques, caused by the infiltration of B cells into the brain parenchyma, could be reduced by the therapeutic depletion of B cells; this could retard disease progression in mice (Kim et al., 2021). Macrophages (microglia), which are the resident innate immune cells of the CNS, are pivotal in maintaining immune defense and homeostasis (Madore et al., 2020). In AD, it is clued that CD8 cells was the key immune cells (Li et al., 2022). Eosinophils, M0 macrophages, M1 macrophages and CD8 cells were relevant to AD, especially M1 macrophages (Liu et al., 2022). What’s more, it was confirmed that CD8^+^ T-cells could infiltrate the aged and AD brain, in order that brain CD8^+^ T-cells maybe directly lead to neuronal dysfunction in modulating synaptic plasticity (Unger et al., 2020). In addition, contributing to neuroinflammation associated to AD, the infiltrated peripheral Th1 immune cells are relevant to the M1 microglia activation in brain (Wang et al., 2019). Overall, the immune infiltration of AD was related to CD4 cells, CD8 cells, and macrophages. Herein, the correlation between *AGAP3* and immune infiltrating cells was assessed, revealing significant changes in immune infiltrating cells, such as follicular helper T cells, naïve CD4 T cells, naïve B cells, memory B cells, macrophages M0, macrophages M1, macrophages M2, resting NK cells, activated NK cells, monocytes, neutrophils, eosinophils, and activated mast cells. The results indicated that *AGAP3* may exert a significant effect on AD by influencing immune infiltrating cells, especially CD4 cells, CD8 cells, and macrophages.

The present study does have some limitations that need to be considered. First, the GEO database lacks complete clinical data, and has shortcomings in terms of the analysis of clinical characteristics; thus, the data have low statistical power. Second, the interactions between factors could not be identified. Hence, further attempts should be made to identify these and verify the relationships between *AGAP3* and immune infiltrating cells. More clinical evidence on AD patients must be obtained about this study for further subgroup analysis. However, further experimental techniques, such as real-time PCR and western blot, need to be applied to elucidate the role of hub genes and the underlying mechanisms of AD. Future studies should not only focus on studying the molecular, organismal, and cellular levels of *AGAP3* and also explore the effects of *AGAP3* on immune infiltrating cells in relation to AD.

## Conclusion

Overall, this study provides a better understanding of immune-related genes related to AD through comprehensive analyses of gene datasets from the GEO database. These immune-related genes could provide new insights for the early diagnosis of AD. This study also highlights the role of *AGAP3* and offers directions for further biological studies on AD development and immunity.

## Data availability statement

The original contributions presented in this study are included in the article/Supplementary material, further inquiries can be directed to the corresponding author.

## Ethics statement

Ethical review and approval was not required for the study on human participants in accordance with the local legislation and institutional requirements. Written informed consent from the patients/participants or patients/participants’ legal guardian/next of kin was not required to participate in this study in accordance with the national legislation and the institutional requirements.

## Author contributions

KZ and HZ: study design, data acquisition, analysis, completed drawing, and writing and editing of the manuscript. YYW, JZL, and XZL: data acquisition and analysis. JYL: data acquisition, analysis, interpretation, and critical revision of the manuscript. All authors have read and approved the final manuscript.

## Footnotes

1
https://www.ncbi.nlm.nih.gov/geo/

2
https://www.gsea-msigdb.org/gsea/index.jsp

3
https://www.immport.org/shared/home

4
https://cn.string-db.org/

5
http://starbase.sysu.edu.cn/

6
http://mirdb.org/

7
http://mirwalk.umm.uni-heidelberg.de/

8
https://www.r-project.org/
