# Supplementary material for: Integrated analysis and identification of hub genes as novel biomarkers for Alzheimer’s disease
Source: Front Aging Neurosci. 2022 Aug 30;14:901972. doi: 10.3389/fnagi.2022.901972 (PMC9468260; doi:10.3389/fnagi.2022.901972)
Supplement: Supplementary file 1 [file Data_Sheet_1.pdf]

GSE5281

Accession	Title	Source name	Sample Amount	Bio-Source Name	Organism	Organ/Tissue Type	Organ Region	Cell Type	Ethnicity	Developm	Disease S	Sex	Genetic V	Age	Sample amount	Bio-source name	Organism	Organ/tis	Organ region	Cell type	Ethnicity	Developm	Disease state	Sex	Genetic variatio	Age
GSM119615	EC contrc	brain, Entorhinal Cortex	10 ug	EC control 1	Human	brain	Entorhinal Cortex	layer III neurons	Caucasia	Adult	normal	male	None	63 years												
GSM119616	EC contrc	brain, Entorhinal Cortex	10 ug	EC control 2	Human	brain	Entorhinal Cortex	layer III neurons	Caucasia	Adult	normal	male	None	85 years												
GSM119617	EC contrc	brain, Entorhinal Cortex	10 ug	EC control 3	Human	brain	Entorhinal Cortex	layer III neurons	Caucasia	Adult	normal	male	None	80 years												
GSM119618	EC contrc	brain, Entorhinal Cortex	10 ug	EC control 4	Human	brain	Entorhinal Cortex	layer III neurons	Caucasia	Adult	normal	male	None	80 years												
GSM119619	EC contrc	brain, Entorhinal Cortex	10 ug	EC control 5	Human	brain	Entorhinal Cortex	layer III neurons	Caucasia	Adult	normal	female	None	102 years												
GSM119620	EC contrc	brain, Entorhinal Cortex	10 ug	EC control 6	Human	brain	Entorhinal Cortex	layer III neurons	Caucasia	Adult	normal	male	None	79 years												
GSM119621	EC contrc	brain, Entorhinal Cortex	10 ug	EC control 7	Human	brain	Entorhinal Cortex	layer III neurons	Caucasia	Adult	normal	male	None	76 years												
GSM119622	EC contrc	brain, Entorhinal Cortex	10 ug	EC control 8	Human	brain	Entorhinal Cortex	layer III neurons	Caucasia	Adult	normal	male	None	83 years												
GSM119623	EC contrc	brain, Entorhinal Cortex	10 ug	EC control 9	Human	brain	Entorhinal Cortex	layer III neurons	Caucasia	Adult	normal	male	None	79 years												
GSM119624	EC contrc	brain, Entorhinal Cortex	10 ug	EC control 10	Human	brain	Entorhinal Cortex	layer III neurons	Caucasia	Adult	normal	female	None	88 years												
GSM119625	EC contrc	brain, Entorhinal Cortex	10 ug	EC control 11	Human	brain	Entorhinal Cortex	layer III neurons	Caucasia	Adult	normal	female	None	82 years												
GSM119626	EC contrc	brain, Entorhinal Cortex	10 ug	EC control 12	Human	brain	Entorhinal Cortex	layer III neurons	Caucasia	Adult	normal	male	None	69 years												
GSM119627	EC contrc	brain, Entorhinal Cortex	10 ug	EC control 13	Human	brain	Entorhinal Cortex	layer III neurons	Caucasia	Adult	normal	male	None	78 years												
GSM119628	HIP contr	brain, hippocampus	10 ug	HIP control 1	Human	brain	hippocampus	layer III neurons	Caucasia	Adult	normal	male	None	85 days												
GSM119629	HIP contr	brain, hippocampus	10 ug	HIP control 2	Human	brain	hippocampus	layer III neurons	Caucasia	Adult	normal	male	None	80 years												
GSM119630	HIP contr	brain, hippocampus	10 ug	HIP control 3	Human	brain	hippocampus	layer III neurons	Caucasia	Adult	normal	male	None	80 years												
GSM119631	HIP contr	brain, hippocampus	10 ug	HIP control 4	Human	brain	hippocampus	layer III neurons	Caucasia	Adult	normal	female	None	102 years												
GSM119632	HIP contr	brain, hippocampus	10 ug	HIP control 5	Human	brain	hippocampus	layer III neurons	Caucasia	Adult	normal	male	None	63 years												
GSM119633	HIP contr	brain, hippocampus	10 ug	HIP control 6	Human	brain	hippocampus	layer III neurons	Caucasia	Adult	normal	male	None	79 years												
GSM119634	HIP contr	brain, hippocampus	10 ug	HIP control 7	Human	brain	hippocampus	layer III neurons	Caucasia	Adult	normal	male	None	76 years												
GSM119635	HIP contr	brain, hippocampus	10 ug	HIP control 8	Human	brain	hippocampus	layer III neurons	Caucasia	Adult	normal	male	None	83 years												
GSM119636	HIP contr	brain, hippocampus	10 ug	HIP control 9	Human	brain	hippocampus	layer III neurons	Caucasia	Adult	normal	male	None	79 years												
GSM119637	HIP contr	brain, hippocampus	10 ug	HIP control 10	Human	brain	hippocampus	layer III neurons	Caucasia	Adult	normal	female	None	88 years												
GSM119638	HIP contr	brain, hippocampus	10 ug	HIP control 11	Human	brain	hippocampus	layer III neurons	Caucasia	Adult	normal	female	None	73 years												
GSM119639	HIP contr	brain, hippocampus	10 ug	HIP control 12	Human	brain	hippocampus	layer III neurons	Caucasia	Adult	normal	male	None	69 years												
GSM119640	HIP contr	brain, hippocampus	10 ug	HIP control 13	Human	brain	hippocampus	layer III neurons	Caucasia	Adult	normal	male	None	78 years												
GSM119641	MTG cont	brain, Medial Temporal Gyrus	10 ug	MTG control 1	Human	brain	Medial Temporal Gyrus	layer III neurons	Caucasia	Adult	normal	male	None	85 years												
GSM119642	MTG cont	brain, Medial Temporal Gyrus	10 ug	MTG control 2	Human	brain	Medial Temporal Gyrus	layer III neurons	Caucasia	Adult	normal	male	None	80 years												
GSM119643	MTG cont	brain, Medial Temporal Gyrus	10 ug	MTG control 3	Human	brain	Medial Temporal Gyrus	layer III neurons	Caucasia	Adult	normal	female	None	102 years												
GSM119644	MTG cont	brain, Medial Temporal Gyrus	10 ug	MTG control 4	Human	brain	Medial Temporal Gyrus	layer III neurons	Caucasia	Adult	normal	male	None	63 years												
GSM119645	MTG cont	brain, Medial Temporal Gyrus	10 ug	MTG control 5	Human	brain	Medial Temporal Gyrus	layer III neurons	Caucasia	Adult	normal	male	None	79 years												
GSM119646	MTG cont	brain, Medial Temporal Gyrus	10 ug	MTG control 6	Human	brain	Medial Temporal Gyrus	layer III neurons	Caucasia	Adult	normal	male	None	83 years												
GSM119647	MTG cont	brain, Medial Temporal Gyrus	10 ug	MTG control 7	Human	brain	Medial Temporal Gyrus	layer III neurons	Caucasia	Adult	normal	male	None	79 years												
GSM119648	MTG cont	brain, Medial Temporal Gyrus	10 ug	MTG control 8	Human	brain	Medial Temporal Gyrus	layer III neurons	Caucasia	Adult	normal	female	None	88 years												
GSM119649	MTG cont	brain, Medial Temporal Gyrus	10 ug	MTG control 9	Human	brain	Medial Temporal Gyrus	layer III neurons	Caucasia	Adult	normal	female	None	82 years												
GSM119650	MTG cont	brain, Medial Temporal Gyrus	10 ug	MTG control 10	Human	brain	Medial Temporal Gyrus	layer III neurons	Caucasia	Adult	normal	female	None	73 years												
GSM119651	MTG cont	brain, Medial Temporal Gyrus	10 ug	MTG control 11	Human	brain	Medial Temporal Gyrus	layer III neurons	Caucasia	Adult	normal	male	None	69 years												
GSM119652	MTG cont	brain, Medial Temporal Gyrus	10 ug	MTG control 12	Human	brain	Medial Temporal Gyrus	layer III neurons	Caucasia	Adult	normal	male	None	78 years												
GSM119653	PC contrc	brain, Posterior Cingulate													10 ug	PC control 1	Human	brain	Posterior Cingulate	layer III neurons	Caucasia	Adult	normal	male	None	85 years
GSM119654	PC contrc	brain, Posterior Cingulate													10 ug	PC control 2	Human	brain	Posterior Cingulate	layer III neurons	Caucasia	Adult	normal	male	None	80 years
GSM119655	PC contrc	brain, Posterior Cingulate													10 ug	PC control 3	Human	brain	Posterior Cingulate	layer III neurons	Caucasia	Adult	normal	female	None	102 years
GSM119656	PC contrc	brain, Posterior Cingulate													10 ug	PC control 4	Human	brain	Posterior Cingulate	layer III neurons	Caucasia	Adult	normal	male	None	63 years
GSM119657	PC contrc	brain, Posterior Cingulate													10 ug	PC control 5	Human	brain	Posterior Cingulate	layer III neurons	Caucasia	Adult	normal	male	None	79 years
GSM119658	PC contrc	brain, Posterior Cingulate													10 ug	PC control 6	Human	brain	Posterior Singulate	layer III neurons	Caucasia	Adult	normal	male	None	76 years
GSM119659	PC contrc	brain, Posterior cingulate													10 ug	PC control 7	Human	brain	Posterior cingulate	layer III neurons	Caucasia	Adult	normal	male	None	83 years
GSM119660	PC contrc	brain, Posterior cingulate													10 ug	PC control 8	Human	brain	Posterior cingulate	layer III neurons	Caucasia	Adult	normal	male	None	79 years
GSM119661	PC contrc	brain, Posterior cingulate													10 ug	PC control 9	Human	brain	Posterior cingulate	layer III neurons	Caucasia	Adult	normal	female	None	88 years
GSM119662	PC contrc	brain, Posterior cingulate													10 ug	PC control 10	Human	brain	Posterior cingulate	layer III neurons	Caucasia	Adult	normal	female	None	82 years
GSM119663	PC contrc	brain, Posterior cingulate													10 ug	PC control 11	Human	brain	Posterior cingulate	layer III neurons	Caucasia	Adult	normal	female	None	73 years
GSM119664	PC contrc	brain, Posterior cingulate													10 ug	PC control 12	Human	brain	Posterior cingulate	layer III neurons	Caucasia	Adult	normal	male	None	69 years
GSM119665	PC contrc	brain, Posterior cingulate													10 ug	PC control 13	Human	brain	Posterior cingulate	layer III neurons	Caucasia	Adult	normal	male	None	78 years
GSM119666	SFG cont	brain, Superior Frontal Gyrus	10 ug	SFG control 1	Human	brain	Superior Frontal Gyrus	layer III neurons	Caucasia	Adult	normal	male	None	79 years												
GSM119667	SFG cont	brain, Superior Frontal Gyrus	10 ug	SFG control 2	Human	brain	Superior Frontal Gyrus	layer III neurons	Caucasia	Adult	normal	female	None	88 years												
GSM119668	SFG cont	brain, Superior Frontal Gyrus	10 ug	SFG control 3	Human	brain	Superior Frontal Gyrus	layer III neurons	Caucasia	Adult	normal	female	None	82 years												
GSM119669	SFG cont	brain, Superior Frontal Gyrus	10 ug	SFG control 4	Human	brain	Superior Frontal Gyrus	layer III neurons	Caucasia	Adult	normal	female	None	73 years												
GSM119670	SFG cont	brain, Superior Frontal Gyrus	10 ug	SFG control 5	Human	brain	Superior Frontal Gyrus	layer III neurons	Caucasia	Adult	normal	female	None	102 years												
GSM119671	SFG cont	brain, Superior Frontal Gyrus	10 ug	SFG control 6	Human	brain	Superior Frontal Gyrus	layer III neurons	Caucasia	Adult	normal	male	None	63 years												
GSM119672	SFG cont	brain, Superior Frontal Gyrus	10 ug	SFG control 7	Human	brain	Superior Frontal Gyrus	layer III neurons	Caucasia	Adult	normal	male	None	79 years												
GSM119673	SFG cont	brain, Superior Frontal Gyrus	10 ug	SFG control 8	Human	brain	Superior Frontal Gyrus	layer III neurons	Caucasia	Adult	normal	male	None	76 years												
GSM119674	SFG cont	brain, Superior Frontal Gyrus	10 ug	SFG control 9	Human	brain	Superior Frontal Gyrus	layer III neurons	Caucasia	Adult	normal	male	None	83 years												
GSM119675	SFG cont	brain, Superior Frontal Gyrus	10 ug	SFG control 10	Human	brain	Superior Frontal Gyrus	layer III neurons	Caucasia	Adult	normal	male	None	69 years												
GSM119676	SFG cont	brain, Superior Frontal Gyrus	10 ug	SFG control 11	Human	brain	Superior Frontal Gyrus	layer III neurons	Caucasia	Adult	normal	male	None	78 years												
GSM119677	VCX cont	brain, Primary Visual Cortex	10 ug	VCX control 1	Human	brain	Primary Visual Cortex	layer III neurons	Caucasia	Adult	normal	male	None	85 years												
GSM119678	VCX cont	brain, Primary Visual Cortex	10 ug	VCX control 2	Human	brain	Primary Visual Cortex	layer III neurons	Caucasia	Adult	normal	male	None	80 years												
GSM119679	VCX cont	brain, Primary Visual Cortex	10 ug	VCX control 3	Human	brain	Primary Visual Cortex	layer III neurons	Caucasia	Adult	normal	male	None	63 years												
GSM119680	VCX cont	brain, Primary Visual Cortex	10 ug	VCX control 4	Human	brain	Primary Visual Cortex	layer III neurons	Caucasia	Adult	normal	male	None	79 years												
GSM119681	VCX cont	brain, Primary Visual Cortex	10 ug	VCX control 5	Human	brain	Primary Visual Cortex	layer III neurons	Caucasia	Adult	normal	male	None	76 years												
GSM119682	VCX cont	brain, Primary Visual Cortex	10 ug	VCX control 6	Human	brain	Primary Visual Cortex	layer III neurons	Caucasia	Adult	normal	male	None	83 years												
GSM119683	VCX cont	brain, Primary Visual Cortex	10 ug	VCX control 7	Human	brain	Primary Visual Cortex	layer III neurons	Caucasia	Adult	normal	male	None	79 years												
GSM119684	VCX cont	brain, Primary Visual Cortex	10 ug	VCX control 8	Human	brain	Primary Visual Cortex	layer III neurons	Caucasia	Adult	normal	female	None	88 years												
GSM119685	VCX cont	brain, Primary Visual Cortex	10 ug	VCX control 9	Human	brain	Primary Visual Cortex	layer III neurons	Caucasia	Adult	normal	female	None	82 years												
GSM119686	VCX cont	brain, Primary Visual Cortex	10 ug	VCX control 10	Human	brain	Primary Visual Cortex	layer III neurons	Caucasia	Adult	normal	female	None	73 years												
GSM119687	VCX cont	brain, Primary Visual Cortex	10 ug	VCX control 11	Human	brain	Primary Visual Cortex	layer III neurons	Caucasia	Adult	normal	male	None	69 years												
GSM119688	VCX cont	brain, Primary Visual Cortex	10 ug	VCX control 12	Human	brain	Primary Visual Cortex	layer III neurons	Caucasia	Adult	normal	male	None	78 years												
GSM238763	EC affect	brain, Entorhinal Cortex	10 ng	EC affected 1	Human	brain	Entorhinal Cortex	pyramidal neuron	unknown	Adult	Alzheimei	female	None	82 years												
GSM238790	EC affect	brain, Entorhinal Cortex	10 ng	EC affected 2	Human	brain	Entorhinal Cortex	pyramidal neuron	unknown	Adult	Alzheimei	female	None	86 years												
GSM238791	EC affect	brain, Entorhinal Cortex	10 ug	EC affected 3	Human	brain	Entorhinal Cortex	pyramidal neuron	unknown	Adult	Alzheimei	female	None	93 years												
GSM238792	EC affect	brain, Entorhinal Cortex	10 ng	EC affected 4	Human	brain	Entorhinal Cortex	pyramidal neuron	unknown	Adult	Alzheimei	male	None	84 years												
GSM238793	EC affect	brain, Entorhinal Cortex	10 ng	EC affected 5	Human	brain	Entorhinal Cortex	pyramidal neuron	unknown	Adult	Alzheimei	female	None	79 years												
GSM238794	EC affect	brain, Entorhinal Cortex	10 ng	EC affected 6	Human	brain	Entorhinal Cortex	pyramidal neuron	unknown	Adult	Alzheimei	female	None	78 years												
GSM238795	EC affect	brain, Entorhinal Cortex	10 ng	EC affected 7	Human	brain	Entorhinal Cortex	pyramidal neuron	unknown	Adult	Alzheimei	female	None	91 years												
GSM238796	EC affect	brain, Entorhinal Cortex													10 ng	EC affected 8	Human	brain	Entorhinal Cortex	pyramidal neuron	unknown	Adult	Alzheimer's Disease	male	None	86 years
GSM238797	EC affect	brain, Entorhinal Cortex													10 ng	EC affected 9	Human	brain	Entorhinal Cortex	pyramidal neuron	unknown	Adult	Alzheimer's Disease	male	None	97 years
GSM238798	EC affect	brain, Entorhinal Cortex	10 ng	EC affected 10	Human	brain	Entorhinal Cortex	pyramidal neuron	unknown	Adult	Alzheimei	male	None	80 years												
GSM238799	HIP affec	brain, Hippocampus													10 ng	HIP affected 1	Human	brain	hippocampus	pyramidal neuron	unknown	Adult	Alzheimer's Disease	male	None	72 years
GSM238800	HIP affec	brain, Hippocampus													10 ng	HIP affected 2	Human	brain	hippocampus	pyramidal neuron	unknown	Adult	Alzheimer's Disease	female	None	73 years
GSM238801	HIP affec	brain, Hippocampus													10 ng	HIP affected 3	Human	brain	hippocampus	pyramidal neuron	unknown	Adult	Alzheimer's Disease	male	None	75 years
GSM238802	HIP affec	brain, Hippocampus													10 ng	HIP affected 4	Human	brain	hippocampus	pyramidal neuron	unknown	Adult	Alzheimer's Disease	female	None	70 years
GSM238803	HIP affec	brain, Hippocampus													10 ng	HIP affected 5	Human	brain	hippocampus	pyramidal neuron	unknown	Adult	Alzheimer's Disease	female	None	85 years
GSM238804	HIP affec	brain, Hippocampus													10 ng	HIP affected 6	Human	brain	hippocampus	pyramidal neuron	unknown	Adult	Alzheimer's Disease	female	None	77 years
GSM238805	HIP affec	brain, Hippocampus													10 ng	HIP affected 7	Human	brain	hippocampus	pyramidal neuron	unknown	Adult	Alzheimer's Disease	male	None	79 years
GSM238806	HIP affec	brain, Hippocampus													10 ng	HIP affected 8	Human	brain	hippocampus	pyramidal neuron	unknown	Adult	Alzheimer's Disease	male	None	88 years
GSM238807	HIP affec	brain, Hippocampus													10 ng	HIP affected 9	Human	brain	hippocampus	pyramidal neuron	unknown	Adult	Alzheimer's Disease	male	None	81 years
GSM238808	HIP affec	brain, Hippocampus													10 ng	HIP affected 10	Human	brain	hippocampus	layer III neurons	Caucasia	Adult	Alzheimer's Disease	male	None	78 years
GSM238809	MTG affe	brain, Middle temporal gyrus													10 ng	MTG affected 1	Human	brain	Medial Temporal Gyrus	pyramidal neuron	unknown	Adult	Alzheimer's Disease	female	None	73 years
GSM238810	MTG affe	brain, Middle temporal gyrus													10 ng	MTG affected 2	Human	brain	Medial Temporal Gyrus	pyramidal neuron	unknown	Adult	Alzheimer's Disease	male	None	81 years
GSM238811	MTG affe	brain, Middle temporal gyrus													10 ng	MTG affected 3	Human	brain	Medial Temporal Gyrus	pyramidal neuron	unknown	Adult	Alzheimer's Disease	male	None	72 years
GSM238812	MTG affe	brain, Middle temporal gyrus													10 ng	MTG affected 4	Human	brain	Medial Temporal Gyrus	pyramidal neuron	unknown	Adult	Alzheimer's Disease	male	None	75 years
GSM238813	MTG affe	brain, Middle temporal gyrus													10 ng	MTG affected 5	Human	brain	Medial Temporal Gyrus	pyramidal neuron	unknown	Adult	Alzheimer's Disease	male	None	78 years
GSM238815	MTG affe	brain, Middle temporal gyrus													10 ng	MTG affected 6	Human	brain	Medial Temporal Gyrus	pyramidal neuron	unknown	Adult	Alzheimer's Disease	male	None	75 years
GSM238816	MTG affe	brain, Middle temporal gyrus													10 ng	MTG affected 7	Human	brain	Medial Temporal Gyrus	pyramidal neuron	unknown	Adult	Alzheimer's Disease	male	None	87 years
GSM238817	MTG affe	brain, Middle temporal gyrus													10 ng	MTG affected 8	Human	brain	Medial Temporal Gyrus	pyramidal neuron	unknown	Adult	Alzheimer's Disease	female	None	>90 years
GSM238818	MTG affe	brain, Middle temporal gyrus													10 ng	MTG affected 9	Human	brain	Medial Temporal Gyrus	pyramidal neuron	unknown	Adult	Alzheimer's Disease	male	None	68 years
GSM238819	MTG affe	brain, Middle temporal gyrus													10 ng	MTG affected 10	Human	brain	Medial Temporal Gyrus	pyramidal neuron	unknown	Adult	Alzheimer's Disease	female	None	81 years
GSM238820	MTG affe	brain, Middle temporal gyrus													10 ng	MTG affected 11	Human	brain	Medial Temporal Gyrus	pyramidal neuron	unknown	Adult	Alzheimer's Disease	female	None	85 years
GSM238821	MTG affe	brain, Middle temporal gyrus													10 ng	MTG affected 12	Human	brain	Medial Temporal Gyrus	pyramidal neuron	unknown	Adult	Alzheimer's Disease	male	None	79 years
GSM238822	MTG affe	brain, Middle temporal gyrus													10 ng	MTG affected 13	Human	brain	Medial Temporal Gyrus	layer III neurons	Caucasia	Adult	Alzheimer's Disease	female	None	82 years
GSM238823	MTG affe	brain, Middle temporal gyrus													10 ng	MTG affected 14	Human	brain	Medial Temporal Gyrus	layer III neurons	Caucasia	Adult	Alzheimer's Disease	male	None	88 years
GSM238824	MTG affe	brain, Middle temporal gyrus													10 ng	MTG affected 15	Human	brain	Medial Temporal Gyrus	layer III neurons	Caucasia	Adult	Alzheimer's Disease	male	None	72 years
GSM238825	MTG affe	brain, Middle temporal gyrus													10 ng	MTG affected 16	Human	brain	Medial Temporal Gyrus	layer III neurons	Caucasia	Adult	Alzheimer's Disease	female	None	80 years
GSM238826	PC affect	brain, Posterior cingulate cortex													10 ng	PC affected 1	Human	brain	Posterior Cingulate	pyramidal neuron	unknown	Adult	Alzheimer's Disease	female	None	73 years
GSM238827	PC affect	brain, Posterior cingulate cortex	10 ng	PC affected 2	Human	brain	Posterior Singulate	pyramidal neuron	unknown	Adult	Alzheimei	male	None	81 years												
GSM238834	PC affect	brain, Posterior cingulate cortex	10 ng	PC affected 3	Human	brain	Posterior Singulate	pyramidal neuron	unknown	Adult	Alzheimei	male	None	78 years												
GSM238835	PC affect	brain, Posterior cingulate cortex	10 ng	PC affected 4	Human	brain	Posterior Singulate	pyramidal neuron	unknown	Adult	Alzheimei	male	None	75 years												
GSM238837	PC affect	brain, Posterior cingulate cortex	10 ng	PC affected 5	Human	brain	Posterior Singulate	pyramidal neuron	unknown	Adult	Alzheimei	male	None	68 years												
GSM238838	PC affect	brain, Posterior cingulate cortex	10 ng	PC affected 6	Human	brain	Posterior Singulate	pyramidal neuron	unknown	Adult	Alzheimei	female	None	70.8 years												
GSM238839	PC affect	brain, Posterior cingulate cortex	10 ng	PC affected 7	Human	brain	Posterior Singulate	pyramidal neuron	unknown	Adult	Alzheimei	female	None	85 years												
GSM238840	PC affect	brain, Posterior cingulate cortex	10 ng	PC affected 8	Human	brain	Posterior Singulate	pyramidal neuron	unknown	Adult	Alzheimei	male	None	79 years												
GSM238841	PC affect	brain, Posterior cingulate cortex	10 ng	PC affected 9	Human	brain	Posterior Singulate	pyramidal neuron	unknown	Adult	Alzheimei	male	None	88 years												
GSM238842	SFG affe	brain, Superior frontal gyrus	10 ng	SFG affected 1	Human	brain	Superior Frontal Gyrus	pyramidal neuron	unknown	Adult	Alzheimei	female	None	73 years												
GSM238843	SFG affe	brain, Superior frontal gyrus	10 ng	SFG affected 2	Human	brain	Superior Frontal Gyrus	pyramidal neuron	unknown	Adult	Alzheimei	male	None	81 years												
GSM238844	SFG affe	brain, Superior frontal gyrus	10 ng	SFG affected 3	Human	brain	Superior Frontal Gyrus	pyramidal neuron	unknown	Adult	Alzheimei	male	None	72 years												
GSM238845	SFG affe	brain, Superior frontal gyrus	10 ng	SFG affected 4	Human	brain	Superior Frontal Gyrus	pyramidal neuron	unknown	Adult	Alzheimei	male	None	75 years												

GSM238846	SFG	affe	brain, Superior frontal gyrus	10 ng	SFG	affected	5	Human	brain	Superior Frontal Gyrus	pyramidal neuron	unknown	Adult	Alzheimer	male	None	78 years														
GSM238847	SFG	affe	brain, Superior frontal gyrus	10 ng	SFG	affected	6	Human	brain	Superior Frontal Gyrus	pyramidal neuron	unknown	Adult	Alzheimer	male	None	75 years														
GSM238848	SFG	affe	brain, Superior frontal gyrus	10 ng	SFG	affected	7	Human	brain	Superior Frontal Gyrus	pyramidal neuron	unknown	Adult	Alzheimer	male	None	87 years														
GSM238851	SFG	affe	brain, Superior frontal gyrus	10 ng	SFG	affected	8	Human	brain	Superior Frontal Gyrus	pyramidal neuron	unknown	Adult	Alzheimer	female	None	95 years														
GSM238854	SFG	affe	brain, Superior frontal gyrus	10 ng	SFG	affected	9	Human	brain	Superior Frontal Gyrus	pyramidal neuron	unknown	Adult	Alzheimer	male	None	68 years														
GSM238855	SFG	affe	brain, Superior frontal gyrus	10 ng	SFG	affected	10	Human	brain	Superior Frontal Gyrus	pyramidal neuron	unknown	Adult	Alzheimer	female	None	95 years														
GSM238856	SFG	affe	brain, Superior frontal gyrus	10 ng	SFG	affected	11	Human	brain	Superior Frontal Gyrus	pyramidal neuron	unknown	Adult	Alzheimer	female	None	70.8 years														
GSM238857	SFG	affe	brain, Superior frontal gyrus	10 ng	SFG	affected	12	Human	brain	Superior Frontal Gyrus	pyramidal neuron	unknown	Adult	Alzheimer	female	None	85 years														
GSM238858	SFG	affe	brain, Superior frontal gyrus	10 ng	SFG	affected	13	Human	brain	Superior Frontal Gyrus	pyramidal neuron	unknown	Adult	Alzheimer	female	None	83 years														
GSM238860	SFG	affe	brain, Superior frontal gyrus	10 ng	SFG	affected	14	Human	brain	Superior Frontal Gyrus	pyramidal neuron	unknown	Adult	Alzheimer	female	None	77 years														
GSM238861	SFG	affe	brain, Superior frontal gyrus	10 ng	SFG	affected	15	Human	brain	Superior Frontal Gyrus	pyramidal neuron	unknown	Adult	Alzheimer	female	None	83 years														
GSM238862	SFG	affe	brain, Superior frontal gyrus	10 ng	SFG	affected	16	Human	brain	Superior Frontal Gyrus	pyramidal neuron	unknown	Adult	Alzheimer	male	None	68 years														
GSM238863	SFG	affe	brain, Superior frontal gyrus	10 ng	SFG	affected	17	Human	brain	Superior Frontal Gyrus	pyramidal neuron	unknown	Adult	Alzheimer	male	None	79 years														
GSM238864	SFG	affe	brain, Superior frontal gyrus	10 ng	SFG	affected	18	Human	brain	Superior Frontal Gyrus	pyramidal neuron	unknown	Adult	Alzheimer	female	None	82 years														
GSM238865	SFG	affe	brain, Superior frontal gyrus															10 ng	SFG	affected	19	Human	brain	Superior Frontal Gyrus	pyramidal neuron	unknown	Adult	Alzheimer's Disease	male	None	80 years
GSM238867	SFG	affe	brain, Superior frontal gyrus															10 ng	SFG	affected	20	Human	brain	Superior Frontal Gyrus	pyramidal neuron	unknown	Adult	Alzheimer's Disease	male	None	88 years
GSM238868	SFG	affe	brain, Superior frontal gyrus															10 ng	SFG	affected	21	Human	brain	Superior Frontal Gyrus	pyramidal neuron	unknown	Adult	Alzheimer's Disease	male	None	74 years
GSM238870	SFG	affe	brain, Superior frontal gyrus															10 ng	SFG	affected	22	Human	brain	Superior Frontal Gyrus	pyramidal neuron	unknown	Adult	Alzheimer's Disease	male	None	72 years
GSM238871	SFG	affe	brain, Superior frontal gyrus															10 ng	SFG	affected	23	Human	brain	Superior Frontal Gyrus	layer III neurons	Caucasia	Adult	Alzheimer's Disease	female	None	80 years
GSM238872	VCX	affe	brain, Primary visual cortex	10 ng	VCX	affected	1	Human	brain	Primary Visual Cortex	pyramidal neuron	unknown	Adult	Alzheimer	female	None	73 years														
GSM238873	VCX	affe	brain, Primary visual cortex	10 ng	VCX	affected	2	Human	brain	Primary Visual Cortex	pyramidal neuron	unknown	Adult	Alzheimer	male	None	81 years														
GSM238874	VCX	affe	brain, Primary visual cortex	10 ng	VCX	affected	3	Human	brain	Primary Visual Cortex	pyramidal neuron	unknown	Adult	Alzheimer	male	None	75 years														
GSM238875	VCX	affe	brain, Primary visual cortex	10 ng	VCX	affected	4	Human	brain	Primary Visual Cortex	pyramidal neuron	unknown	Adult	Alzheimer	male	None	78 years														
GSM238877	VCX	affe	brain, Primary visual cortex	10 ng	VCX	affected	5	Human	brain	Primary Visual Cortex	pyramidal neuron	unknown	Adult	Alzheimer	male	None	75 years														
GSM238941	VCX	affe	brain, Primary visual cortex	10 ng	VCX	affected	6	Human	brain	Primary Visual Cortex	pyramidal neuron	unknown	Adult	Alzheimer	male	None	87 years														
GSM238942	VCX	affe	brain, Primary visual cortex	10 ng	VCX	affected	7	Human	brain	Primary Visual Cortex	pyramidal neuron	unknown	Adult	Alzheimer	female	None	95 years														
GSM238943	VCX	affe	brain, Primary visual cortex	10 ng	VCX	affected	8	Human	brain	Primary Visual Cortex	pyramidal neuron	unknown	Adult	Alzheimer	male	None	68 years														
GSM238944	VCX	affe	brain, Primary visual cortex															10 ng	VCX	affected	9	Human	brain	Primary Visual Cortex	pyramidal neuron	unknown	Adult	Alzheimer's Disease	female	None	>90 years
GSM238945	VCX	affe	brain, Primary visual cortex	10 ng	VCX	affected	10	Human	brain	Primary Visual Cortex	pyramidal neuron	unknown	Adult	Alzheimer	female	None	81.3 years														
GSM238946	VCX	affe	brain, Primary visual cortex															10 ng	VCX	affected	11	Human	brain	Primary Visual Cortex	pyramidal neuron	unknown	Adult	Alzheimer's Disease	female	None	81 years
GSM238947	VCX	affe	brain, Primary visual cortex															10 ng	VCX	affected	12	Human	brain	Primary Visual Cortex	pyramidal neuron	unknown	Adult	Alzheimer's Disease	female	None	85 years
GSM238948	VCX	affe	brain, Primary visual cortex	10 ng	VCX	affected	13	Human	brain	Primary Visual Cortex	pyramidal neuron	unknown	Adult	Alzheimer	male	None	79 years														
GSM238949	VCX	affe	brain, Primary visual cortex															10 ng	VCX	affected	14	Human	brain	Primary Visual Cortex	pyramidal neuron	unknown	Adult	Alzheimer's Disease	male	None	79 years
GSM238951	VCX	affe	brain, Primary visual cortex															10 ng	VCX	affected	15	Human	brain	Primary Visual Cortex	pyramidal neuron	unknown	Adult	Alzheimer's Disease	female	None	82 years
GSM238952	VCX	affe	brain, Primary visual cortex															10 ng	VCX	affected	16	Human	brain	Primary Visual Cortex	pyramidal neuron	unknown	Adult	Alzheimer's Disease	male	None	88 years
GSM238953	VCX	affe	brain, Primary visual cortex															10 ng	VCX	affected	17	Human	brain	Primary Visual Cortex	pyramidal neuron	unknown	Adult	Alzheimer's Disease	male	None	74 years
GSM238955	VCX	affe	brain, Primary visual cortex															10 ng	VCX	affected	18	Human	brain	Primary Visual Cortex	layer III neurons	Caucasia	Adult	Alzheimer's Disease	male	None	72 years
GSM238963	VCX	affe	brain, Primary visual cortex															10 ng	VCX	affected	19	Human	brain	Primary Visual Cortex	layer III neurons	Caucasia	Adult	Alzheimer's Disease	female	None	80 years

GSE28146

Accession	Title	Source name	Sex	Age	Disease status
GSM697308	Control 976	laser capture CA1 tissue gray matter from formalin fixed, paraffin embedded specimen	Male	85 years old	Control
GSM697309	Control 1003	laser capture CA1 tissue gray matter from formalin fixed, paraffin embedded specimen	Male	80 years old	Control
GSM697310	Control 1008	laser capture CA1 tissue gray matter from formalin fixed, paraffin embedded specimen	Female	92 years old	Control
GSM697311	Control 1012	laser capture CA1 tissue gray matter from formalin fixed, paraffin embedded specimen	Male	80 years old	Control
GSM697312	Control 1015	laser capture CA1 tissue gray matter from formalin fixed, paraffin embedded specimen	Male	75 years old	Control
GSM697313	Control 1018	laser capture CA1 tissue gray matter from formalin fixed, paraffin embedded specimen	Female	97 years old	Control
GSM697314	Control 1030	laser capture CA1 tissue gray matter from formalin fixed, paraffin embedded specimen	Male	95 years old	Control
GSM697315	Control 1040	laser capture CA1 tissue gray matter from formalin fixed, paraffin embedded specimen	Male	87 years old	Control
GSM697316	Incipient 715	laser capture CA1 tissue gray matter from formalin fixed, paraffin embedded specimen	Female	101 years old	Incipient
GSM697317	Incipient 720	laser capture CA1 tissue gray matter from formalin fixed, paraffin embedded specimen	Female	95 years old	Incipient
GSM697318	Incipient 994	laser capture CA1 tissue gray matter from formalin fixed, paraffin embedded specimen	Female	83 years old	Incipient
GSM697319	Incipient 1019	laser capture CA1 tissue gray matter from formalin fixed, paraffin embedded specimen	Male	88 years old	Incipient
GSM697320	Incipient 1029	laser capture CA1 tissue gray matter from formalin fixed, paraffin embedded specimen	Female	91 years old	Incipient
GSM697321	Incipient 1034	laser capture CA1 tissue gray matter from formalin fixed, paraffin embedded specimen	Male	88 years old	Incipient
GSM697322	Incipient 1043	laser capture CA1 tissue gray matter from formalin fixed, paraffin embedded specimen	Female	97 years old	Incipient
GSM697323	Moderate 826	laser capture CA1 tissue gray matter from formalin fixed, paraffin embedded specimen	Female	85 years old	Moderate
GSM697324	Moderate 832	laser capture CA1 tissue gray matter from formalin fixed, paraffin embedded specimen	Female	89 years old	Moderate
GSM697325	Moderate 856	laser capture CA1 tissue gray matter from formalin fixed, paraffin embedded specimen	Female	83 years old	Moderate
GSM697326	Moderate 965	laser capture CA1 tissue gray matter from formalin fixed, paraffin embedded specimen	Female	82 years old	Moderate
GSM697327	Moderate 1020	laser capture CA1 tissue gray matter from formalin fixed, paraffin embedded specimen	Female	79 years old	Moderate
GSM697328	Moderate 1025	laser capture CA1 tissue gray matter from formalin fixed, paraffin embedded specimen	Male	81 years old	Moderate
GSM697329	Moderate 1031	laser capture CA1 tissue gray matter from formalin fixed, paraffin embedded specimen	Female	86 years old	Moderate
GSM697330	Moderate 1037	laser capture CA1 tissue gray matter from formalin fixed, paraffin embedded specimen	Male	82 years old	Moderate
GSM697331	Severe 701	laser capture CA1 tissue gray matter from formalin fixed, paraffin embedded specimen	Male	85 years old	Severe
GSM697332	Severe 723	laser capture CA1 tissue gray matter from formalin fixed, paraffin embedded specimen	Female	65 years old	Severe
GSM697333	Severe 807	laser capture CA1 tissue gray matter from formalin fixed, paraffin embedded specimen	Male	93 years old	Severe
GSM697334	Severe 819	laser capture CA1 tissue gray matter from formalin fixed, paraffin embedded specimen	Female	79 years old	Severe
GSM697335	Severe 867	laser capture CA1 tissue gray matter from formalin fixed, paraffin embedded specimen	Female	94 years old	Severe
GSM697336	Severe 872	laser capture CA1 tissue gray matter from formalin fixed, paraffin embedded specimen	Female	79 years old	Severe
GSM697337	Severe 1036	laser capture CA1 tissue gray matter from formalin fixed, paraffin embedded specimen	Female	93 years old	Severe

GSE48350

Accession	Title	Source name	Individual	Brain region	Gender	Characteristics	Braak stage	Apoe genotype	Mmse
GSM300166	PostcentralGyrus_female_91yrs_indiv10	brain, postcentral gyrus, female, 91 years	10, C	postcentral gyrus	female	age (yrs): 91			
GSM300167	SuperiorFrontalGyrus_female_91yrs_indiv10	brain, superior frontal gyrus, female, 91 years	10, C	superior frontal gyrus	female	age (yrs): 91			
GSM300168	Hippocampus_female_96yrs_indiv105	brain, hippocampus, female, 96 years	105, C	hippocampus	female	age (yrs): 96			
GSM300169	Hippocampus_male_82yrs_indiv106	brain, hippocampus, male, 82 years	106, C	hippocampus	male	age (yrs): 82			
GSM300170	Hippocampus_male_84yrs_indiv108	brain, hippocampus, male, 84 years	108, C	hippocampus	male	age (yrs): 84			
GSM300171	Hippocampus_female_87yrs_indiv109	brain, hippocampus, female, 87 years	109, C	hippocampus	female	age (yrs): 87			
GSM300172	Hippocampus_female_82yrs_indiv111	brain, hippocampus, female, 82 years	111, C	hippocampus	female	age (yrs): 82			
GSM300173	EntorhinalCortex_male_45yrs_indiv12	brain, entorhinal cortex, male, 45 years	12, AA	entorhinal cortex	male	age (yrs): 45			
GSM300174	Hippocampus_male_45yrs_indiv12	brain, hippocampus, male, 45 years	12, AA	hippocampus	male	age (yrs): 45			
GSM300175	PostcentralGyrus_male_45yrs_indiv12	brain, postcentral gyrus, male, 45 years	12, AA	postcentral gyrus	male	age (yrs): 45			
GSM300176	SuperiorFrontalGyrus_male_45yrs_indiv12	brain, superior frontal gyrus, male, 45 years	12, AA	superior frontal gyrus	male	age (yrs): 45			
GSM300177	EntorhinalCortex_male_95yrs_indiv14	brain, entorhinal cortex, male, 95 years	14, C	entorhinal cortex	male	age (yrs): 95			
GSM300178	Hippocampus_male_95yrs_indiv14	brain, hippocampus, male, 95 years	14, C	hippocampus	male	age (yrs): 95			
GSM300179	PostcentralGyrus_male_95yrs_indiv14	brain, postcentral gyrus, male, 95 years	14, C	postcentral gyrus	male	age (yrs): 95			
GSM300180	SuperiorFrontalGyrus_male_95yrs_indiv14	brain, superior frontal gyrus, male, 95 years	14, C	superior frontal gyrus	male	age (yrs): 95			
GSM300181	EntorhinalCortex_male_80yrs_indiv15	brain, entorhinal cortex, male, 80 years	15, C	entorhinal cortex	male	age (yrs): 80			
GSM300182	Hippocampus_male_80yrs_indiv15	brain, hippocampus, male, 80 years	15, C	hippocampus	male	age (yrs): 80			
GSM300183	PostcentralGyrus_male_80yrs_indiv15	brain, postcentral gyrus, male, 80 years	15, C	postcentral gyrus	male	age (yrs): 80			
GSM300185	Hippocampus_male_91yrs_indiv16-07	brain, hippocampus, male, 91 years	16-07, C	hippocampus	male	age (yrs): 91			
GSM300186	EntorhinalCortex_female_45yrs_indiv17	brain, entorhinal cortex, female, 45 years	17, C	entorhinal cortex	female	age (yrs): 45			
GSM300187	Hippocampus_female_45yrs_indiv17	brain, hippocampus, female, 45 years	17, C	hippocampus	female	age (yrs): 45			
GSM300188	SuperiorFrontalGyrus_female_45yrs_indiv17	brain, superior frontal gyrus, female, 45 years	17, C	superior frontal gyrus	female	age (yrs): 45			
GSM300189	EntorhinalCortex_female_74yrs_indiv18	brain, entorhinal cortex, female, 74 years	18, C	entorhinal cortex	female	age (yrs): 74			
GSM300190	Hippocampus_female_74yrs_indiv18	brain, hippocampus, female, 74 years	18, C	hippocampus	female	age (yrs): 74			
GSM300191	SuperiorFrontalGyrus_female_74yrs_indiv18	brain, superior frontal gyrus, female, 74 years	18, C	superior frontal gyrus	female	age (yrs): 74			
GSM300192	EntorhinalCortex_female_99yrs_indiv2	brain, entorhinal cortex, female, 99 years	2, C	entorhinal cortex	female	age (yrs): 99			
GSM300193	Hippocampus_female_99yrs_indiv2	brain, hippocampus, female, 99 years	2, C	hippocampus	female	age (yrs): 99			
GSM300194	PostcentralGyrus_female_99yrs_indiv2	brain, postcentral gyrus, female, 99 years	2, C	postcentral gyrus	female	age (yrs): 99			
GSM300195	SuperiorFrontalGyrus_female_99yrs_indiv2	brain, superior frontal gyrus, female, 99 years	2, C	superior frontal gyrus	female	age (yrs): 99			
GSM300196	EntorhinalCortex_female_74yrs_indiv21	brain, entorhinal cortex, female, 74 years	21, C	entorhinal cortex	female	age (yrs): 74			
GSM300197	Hippocampus_female_74yrs_indiv21	brain, hippocampus, female, 74 years	21, C	hippocampus	female	age (yrs): 74			
GSM300198	PostcentralGyrus_female_74yrs_indiv21	brain, postcentral gyrus, female, 74 years	21, C	postcentral gyrus	female	age (yrs): 74			
GSM300199	SuperiorFrontalGyrus_female_74yrs_indiv21	brain, superior frontal gyrus, female, 74 years	21, C	superior frontal gyrus	female	age (yrs): 74			
GSM300200	PostcentralGyrus_female_92yrs_indiv26	brain, postcentral gyrus, female, 92 years	26, C	postcentral gyrus	female	age (yrs): 92			
GSM300201	SuperiorFrontalGyrus_female_92yrs_indiv26	brain, superior frontal gyrus, female, 92 years	26, C	superior frontal gyrus	female	age (yrs): 92			
GSM300202	PostcentralGyrus_female_91yrs_indiv27	brain, postcentral gyrus, female, 91 years	27, C	postcentral gyrus	female	age (yrs): 91			
GSM300203	SuperiorFrontalGyrus_female_91yrs_indiv27	brain, superior frontal gyrus, female, 91 years	27, C	superior frontal gyrus	female	age (yrs): 91			
GSM300204	EntorhinalCortex_male_83yrs_indiv28	brain, entorhinal cortex, male, 83 years	28, C	entorhinal cortex	male	age (yrs): 83			
GSM300205	Hippocampus_male_83yrs_indiv28	brain, hippocampus, male, 83 years	28, C	hippocampus	male	age (yrs): 83			
GSM300206	PostcentralGyrus_male_83yrs_indiv28	brain, postcentral gyrus, male, 83 years	28, C	postcentral gyrus	male	age (yrs): 83			
GSM300207	SuperiorFrontalGyrus_male_83yrs_indiv28	brain, superior frontal gyrus, male, 83 years	28, C	superior frontal gyrus	male	age (yrs): 83			
GSM300208	EntorhinalCortex_male_28yrs_indiv29	brain, entorhinal cortex, male, 28 years	29, C	entorhinal cortex	male	age (yrs): 28			
GSM300209	Hippocampus_male_28yrs_indiv29	brain, hippocampus, male, 28 years	29, C	hippocampus	male	age (yrs): 28			
GSM300210	PostcentralGyrus_male_28yrs_indiv29	brain, postcentral gyrus, male, 28 years	29, C	postcentral gyrus	male	age (yrs): 28			
GSM300211	SuperiorFrontalGyrus_male_28yrs_indiv29	brain, superior frontal gyrus, male, 28 years	29, C	superior frontal gyrus	male	age (yrs): 28			
GSM300212	PostcentralGyrus_female_74yrs_indiv30	brain, postcentral gyrus, female, 74 years	30, C	postcentral gyrus	female	age (yrs): 74			
GSM300213	SuperiorFrontalGyrus_female_74yrs_indiv30	brain, superior frontal gyrus, female, 74 years	30, C	superior frontal gyrus	female	age (yrs): 74			
GSM300214	EntorhinalCortex_male_97yrs_indiv34	brain, entorhinal cortex, male, 97 years	34, C	entorhinal cortex	male	age (yrs): 97			
GSM300215	Hippocampus_male_97yrs_indiv34	brain, hippocampus, male, 97 years	34, C	hippocampus	male	age (yrs): 97			
GSM300216	PostcentralGyrus_male_97yrs_indiv34	brain, postcentral gyrus, male, 97 years	34, C	postcentral gyrus	male	age (yrs): 97			
GSM300217	SuperiorFrontalGyrus_male_97yrs_indiv34	brain, superior frontal gyrus, male, 97 years	34, C	superior frontal gyrus	male	age (yrs): 97			
GSM300218	EntorhinalCortex_female_34yrs_indiv35	brain, entorhinal cortex, female, 34 years	35, AA	entorhinal cortex	female	age (yrs): 34			
GSM300219	Hippocampus_female_34yrs_indiv35	brain, hippocampus, female, 34 years	35, AA	hippocampus	female	age (yrs): 34			
GSM300220	PostcentralGyrus_female_34yrs_indiv35	brain, postcentral gyrus, female, 34 years	35, AA	postcentral gyrus	female	age (yrs): 34			
GSM300221	SuperiorFrontalGyrus_female_34yrs_indiv35	brain, superior frontal gyrus, female, 34 years	35, AA	superior frontal gyrus	female	age (yrs): 34			
GSM300222	EntorhinalCortex_female_74yrs_indiv38	brain, entorhinal cortex, female, 74 years	38, C	entorhinal cortex	female	age (yrs): 74			
GSM300223	Hippocampus_female_74yrs_indiv38	brain, hippocampus, female, 74 years	38, C	hippocampus	female	age (yrs): 74			
GSM300224	PostcentralGyrus_female_74yrs_indiv38	brain, postcentral gyrus, female, 74 years	38, C	postcentral gyrus	female	age (yrs): 74			

GSM300225	SuperiorFrontalGyrus_female_74yrs_indiv38	brain, superior frontal gyrus, female, 74 years	38, C	superior frontal gyrus	female	age (yrs): 74
GSM300226	PostcentralGyrus_female_90yrs_indiv40	brain, postcentral gyrus, female, 90 years	40, C	postcentral gyrus	female	age (yrs): 90
GSM300227	Hippocampus_female_91yrs_indiv40-04	brain, hippocampus, female, 91 years	40-04, C	hippocampus	female	age (yrs): 91
GSM300228	EntorhinalCortex_female_44yrs_indiv42	brain, entorhinal cortex, female, 44 years	42, C	entorhinal cortex	female	age (yrs): 44
GSM300229	SuperiorFrontalGyrus_female_44yrs_indiv42	brain, superior frontal gyrus, female, 44 years	42, C	superior frontal gyrus	female	age (yrs): 44
GSM300230	EntorhinalCortex_female_37yrs_indiv45	brain, entorhinal cortex, female, 37 years	45, C	entorhinal cortex	female	age (yrs): 37
GSM300231	Hippocampus_female_37yrs_indiv45	brain, hippocampus, female, 37 years	45, C	hippocampus	female	age (yrs): 37
GSM300232	PostcentralGyrus_female_37yrs_indiv45	brain, postcentral gyrus, female, 37 years	45, C	postcentral gyrus	female	age (yrs): 37
GSM300233	SuperiorFrontalGyrus_female_37yrs_indiv45	brain, superior frontal gyrus, female, 37 years	45, C	superior frontal gyrus	female	age (yrs): 37
GSM300234	EntorhinalCortex_male_85yrs_indiv46	brain, entorhinal cortex, male, 85 years	46, C	entorhinal cortex	male	age (yrs): 85
GSM300235	Hippocampus_male_85yrs_indiv46	brain, hippocampus, male, 85 years	46, C	hippocampus	male	age (yrs): 85
GSM300236	PostcentralGyrus_male_85yrs_indiv46	brain, postcentral gyrus, male, 85 years	46, C	postcentral gyrus	male	age (yrs): 85
GSM300237	SuperiorFrontalGyrus_male_85yrs_indiv46	brain, superior frontal gyrus, male, 85 years	46, C	superior frontal gyrus	male	age (yrs): 85
GSM300238	EntorhinalCortex_female_70yrs_indiv47	brain, entorhinal cortex, female, 70 years	47, C	entorhinal cortex	female	age (yrs): 70
GSM300239	Hippocampus_female_70yrs_indiv47	brain, hippocampus, female, 70 years	47, C	hippocampus	female	age (yrs): 70
GSM300240	PostcentralGyrus_female_70yrs_indiv47	brain, postcentral gyrus, female, 70 years	47, C	postcentral gyrus	female	age (yrs): 70
GSM300241	SuperiorFrontalGyrus_female_70yrs_indiv47	brain, superior frontal gyrus, female, 70 years	47, C	superior frontal gyrus	female	age (yrs): 70
GSM300242	EntorhinalCortex_female_64yrs_indiv52	brain, entorhinal cortex, female, 64 years	52, C	entorhinal cortex	female	age (yrs): 64
GSM300243	Hippocampus_female_64yrs_indiv52	brain, hippocampus, female, 64 years	52, C	hippocampus	female	age (yrs): 64
GSM300244	PostcentralGyrus_female_64yrs_indiv52	brain, postcentral gyrus, female, 64 years	52, C	postcentral gyrus	female	age (yrs): 64
GSM300245	SuperiorFrontalGyrus_female_64yrs_indiv52	brain, superior frontal gyrus, female, 64 years	52, C	superior frontal gyrus	female	age (yrs): 64
GSM300246	PostcentralGyrus_male_70yrs_indiv53	brain, postcentral gyrus, male, 70 years	53, C	postcentral gyrus	male	age (yrs): 70
GSM300247	SuperiorFrontalGyrus_male_70yrs_indiv53	brain, superior frontal gyrus, male, 70 years	53, C	superior frontal gyrus	male	age (yrs): 70
GSM300248	EntorhinalCortex_female_85yrs_indiv54	brain, entorhinal cortex, female, 85 years	54, C	entorhinal cortex	female	age (yrs): 85
GSM300249	PostcentralGyrus_female_85yrs_indiv54	brain, postcentral gyrus, female, 85 years	54, C	postcentral gyrus	female	age (yrs): 85
GSM300250	SuperiorFrontalGyrus_female_85yrs_indiv54	brain, superior frontal gyrus, female, 85 years	54, C	superior frontal gyrus	female	age (yrs): 85
GSM300251	SuperiorFrontalGyrus_female_91yrs_indiv59	brain, superior frontal gyrus, female, 91 years	59, C	superior frontal gyrus	female	age (yrs): 91
GSM300252	EntorhinalCortex_male_21yrs_indiv66	brain, entorhinal cortex, male, 21 years	66, C	entorhinal cortex	male	age (yrs): 21
GSM300253	PostcentralGyrus_male_21yrs_indiv66	brain, postcentral gyrus, male, 21 years	66, C	postcentral gyrus	male	age (yrs): 21
GSM300254	SuperiorFrontalGyrus_male_21yrs_indiv66	brain, superior frontal gyrus, male, 21 years	66, C	superior frontal gyrus	male	age (yrs): 21
GSM300255	Hippocampus_male_69yrs_indiv67	brain, hippocampus, male, 69 years	67, C	hippocampus	male	age (yrs): 69
GSM300256	PostcentralGyrus_male_69yrs_indiv67	brain, postcentral gyrus, male, 69 years	67, C	postcentral gyrus	male	age (yrs): 69
GSM300258	EntorhinalCortex_male_40yrs_indiv68	brain, entorhinal cortex, male, 40 years	68, C	entorhinal cortex	male	age (yrs): 40
GSM300259	PostcentralGyrus_male_40yrs_indiv68	brain, postcentral gyrus, male, 40 years	68, C	postcentral gyrus	male	age (yrs): 40
GSM300260	SuperiorFrontalGyrus_male_40yrs_indiv68	brain, superior frontal gyrus, male, 40 years	68, C	superior frontal gyrus	male	age (yrs): 40
GSM300261	EntorhinalCortex_male_52yrs_indiv71	brain, entorhinal cortex, male, 52 years	71, C	entorhinal cortex	male	age (yrs): 52
GSM300262	Hippocampus_male_52yrs_indiv71	brain, hippocampus, male, 52 years	71, C	hippocampus	male	age (yrs): 52
GSM300263	PostcentralGyrus_male_52yrs_indiv71	brain, postcentral gyrus, male, 52 years	71, C	postcentral gyrus	male	age (yrs): 52
GSM300264	SuperiorFrontalGyrus_male_52yrs_indiv71	brain, superior frontal gyrus, male, 52 years	71, C	superior frontal gyrus	male	age (yrs): 52
GSM300265	PostcentralGyrus_male_75yrs_indiv72	brain, postcentral gyrus, male, 75 years	72, C	postcentral gyrus	male	age (yrs): 75
GSM300266	SuperiorFrontalGyrus_male_75yrs_indiv72	brain, superior frontal gyrus, male, 75 years	72, C	superior frontal gyrus	male	age (yrs): 75
GSM300267	EntorhinalCortex_male_86yrs_indiv73	brain, entorhinal cortex, male, 86 years	73, C	entorhinal cortex	male	age (yrs): 86
GSM300268	Hippocampus_male_86yrs_indiv73	brain, hippocampus, male, 86 years	73, C	hippocampus	male	age (yrs): 86
GSM300269	PostcentralGyrus_male_86yrs_indiv73	brain, postcentral gyrus, male, 86 years	73, C	postcentral gyrus	male	age (yrs): 86
GSM300270	SuperiorFrontalGyrus_male_86yrs_indiv73	brain, superior frontal gyrus, male, 86 years	73, C	superior frontal gyrus	male	age (yrs): 86
GSM300271	EntorhinalCortex_female_26yrs_indiv76	brain, entorhinal cortex, female, 26 years	76, C	entorhinal cortex	female	age (yrs): 26
GSM300272	Hippocampus_female_26yrs_indiv76	brain, hippocampus, female, 26 years	76, C	hippocampus	female	age (yrs): 26
GSM300273	PostcentralGyrus_female_26yrs_indiv76	brain, postcentral gyrus, female, 26 years	76, C	postcentral gyrus	female	age (yrs): 26
GSM300274	SuperiorFrontalGyrus_female_26yrs_indiv76	brain, superior frontal gyrus, female, 26 years	76, C	superior frontal gyrus	female	age (yrs): 26
GSM300275	EntorhinalCortex_male_20yrs_indiv77	brain, entorhinal cortex, male, 20 years	77, C	entorhinal cortex	male	age (yrs): 20
GSM300276	Hippocampus_male_20yrs_indiv77	brain, hippocampus, male, 20 years	77, C	hippocampus	male	age (yrs): 20
GSM300277	PostcentralGyrus_male_20yrs_indiv77	brain, postcentral gyrus, male, 20 years	77, C	postcentral gyrus	male	age (yrs): 20
GSM300278	SuperiorFrontalGyrus_male_20yrs_indiv77	brain, superior frontal gyrus, male, 20 years	77, C	superior frontal gyrus	male	age (yrs): 20
GSM300279	EntorhinalCortex_male_20yrs_indiv78	brain, entorhinal cortex, male, 20 years	78, C	entorhinal cortex	male	age (yrs): 20
GSM300280	Hippocampus_male_20yrs_indiv78	brain, hippocampus, male, 20 years	78, C	hippocampus	male	age (yrs): 20
GSM300281	PostcentralGyrus_male_20yrs_indiv78	brain, postcentral gyrus, male, 20 years	78, C	postcentral gyrus	male	age (yrs): 20
GSM300282	SuperiorFrontalGyrus_male_20yrs_indiv78	brain, superior frontal gyrus, male, 20 years	78, C	superior frontal gyrus	male	age (yrs): 20
GSM300283	PostcentralGyrus_female_36yrs_indiv79	brain, postcentral gyrus, female, 36 years	79, AA	postcentral gyrus	female	age (yrs): 36
GSM300284	SuperiorFrontalGyrus_female_36yrs_indiv79	brain, superior frontal gyrus, female, 36 years	79, AA	superior frontal gyrus	female	age (yrs): 36
GSM300285	EntorhinalCortex_male_69yrs_indiv8	brain, entorhinal cortex, male, 69 years	8, C	entorhinal cortex	male	age (yrs): 69
GSM300286	Hippocampus_male_69yrs_indiv8	brain, hippocampus, male, 69 years	8, C	hippocampus	male	age (yrs): 69

GSM300287	PostcentralGyrus_male_69yrs_indiv8	brain, postcentral gyrus, male, 69 years	8, C	postcentral gyrus	male	age (yrs): 69			
GSM300288	SuperiorFrontalGyrus_male_69yrs_indiv8	brain, superior frontal gyrus, male, 69 years	8, C	superior frontal gyrus	male	age (yrs): 69			
GSM300289	EntorhinalCortex_female_44yrs_indiv80	brain, entorhinal cortex, female, 44 years	80, AA	entorhinal cortex	female	age (yrs): 44			
GSM300290	Hippocampus_female_44yrs_indiv80	brain, hippocampus, female, 44 years	80, AA	hippocampus	female	age (yrs): 44			
GSM300291	PostcentralGyrus_female_44yrs_indiv80	brain, postcentral gyrus, female, 44 years	80, AA	postcentral gyrus	female	age (yrs): 44			
GSM300292	SuperiorFrontalGyrus_female_44yrs_indiv80	brain, superior frontal gyrus, female, 44 years	80, AA	superior frontal gyrus	female	age (yrs): 44			
GSM300293	EntorhinalCortex_female_48yrs_indiv81	brain, entorhinal cortex, female, 48 years	81, AA	entorhinal cortex	female	age (yrs): 48			
GSM300294	Hippocampus_female_48yrs_indiv81	brain, hippocampus, female, 48 years	81, AA	hippocampus	female	age (yrs): 48			
GSM300295	PostcentralGyrus_female_48yrs_indiv81	brain, postcentral gyrus, female, 48 years	81, AA	postcentral gyrus	female	age (yrs): 48			
GSM300296	SuperiorFrontalGyrus_female_48yrs_indiv81	brain, superior frontal gyrus, female, 48 years	81, AA	superior frontal gyrus	female	age (yrs): 48			
GSM300297	EntorhinalCortex_female_30yrs_indiv82	brain, entorhinal cortex, female, 30 years	82, AA	entorhinal cortex	female	age (yrs): 30			
GSM300298	Hippocampus_female_30yrs_indiv82	brain, hippocampus, female, 30 years	82, AA	hippocampus	female	age (yrs): 30			
GSM300299	SuperiorFrontalGyrus_female_30yrs_indiv82	brain, superior frontal gyrus, female, 30 years	82, AA	superior frontal gyrus	female	age (yrs): 30			
GSM300300	EntorhinalCortex_male_20yrs_indiv83	brain, entorhinal cortex, male, 20 years	83, C	entorhinal cortex	male	age (yrs): 20			
GSM300301	Hippocampus_male_20yrs_indiv83	brain, hippocampus, male, 20 years	83, C	hippocampus	male	age (yrs): 20			
GSM300302	PostcentralGyrus_male_20yrs_indiv83	brain, postcentral gyrus, male, 20 years	83, C	postcentral gyrus	male	age (yrs): 20			
GSM300303	SuperiorFrontalGyrus_male_20yrs_indiv83	brain, superior frontal gyrus, male, 20 years	83, C	superior frontal gyrus	male	age (yrs): 20			
GSM300304	EntorhinalCortex_male_33yrs_indiv84	brain, entorhinal cortex, male, 33 years	84, AA	entorhinal cortex	male	age (yrs): 33			
GSM300305	Hippocampus_male_33yrs_indiv84	brain, hippocampus, male, 33 years	84, AA	hippocampus	male	age (yrs): 33			
GSM300306	PostcentralGyrus_male_33yrs_indiv84	brain, postcentral gyrus, male, 33 years	84, AA	postcentral gyrus	male	age (yrs): 33			
GSM300307	SuperiorFrontalGyrus_male_33yrs_indiv84	brain, superior frontal gyrus, male, 33 years	84, AA	superior frontal gyrus	male	age (yrs): 33			
GSM300308	EntorhinalCortex_male_22yrs_indiv85	brain, entorhinal cortex, male, 22 years	85, C	entorhinal cortex	male	age (yrs): 22			
GSM300309	Hippocampus_male_22yrs_indiv85	brain, hippocampus, male, 22 years	85, C	hippocampus	male	age (yrs): 22			
GSM300310	PostcentralGyrus_male_22yrs_indiv85	brain, postcentral gyrus, male, 22 years	85, C	postcentral gyrus	male	age (yrs): 22			
GSM300311	SuperiorFrontalGyrus_male_22yrs_indiv85	brain, superior frontal gyrus, male, 22 years	85, C	superior frontal gyrus	male	age (yrs): 22			
GSM300312	EntorhinalCortex_male_42yrs_indiv86	brain, entorhinal cortex, male, 42 years	86, AA	entorhinal cortex	male	age (yrs): 42			
GSM300313	Hippocampus_male_42yrs_indiv86	brain, hippocampus, male, 42 years	86, AA	hippocampus	male	age (yrs): 42			
GSM300314	PostcentralGyrus_male_42yrs_indiv86	brain, postcentral gyrus, male, 42 years	86, AA	postcentral gyrus	male	age (yrs): 42			
GSM300315	SuperiorFrontalGyrus_male_42yrs_indiv86	brain, superior frontal gyrus, male, 42 years	86, AA	superior frontal gyrus	male	age (yrs): 42			
GSM300316	EntorhinalCortex_male_45yrs_indiv87	brain, entorhinal cortex, male, 45 years	87, C	entorhinal cortex	male	age (yrs): 45			
GSM300317	Hippocampus_male_45yrs_indiv87	brain, hippocampus, male, 45 years	87, C	hippocampus	male	age (yrs): 45			
GSM300318	PostcentralGyrus_male_45yrs_indiv87	brain, postcentral gyrus, male, 45 years	87, C	postcentral gyrus	male	age (yrs): 45			
GSM300319	SuperiorFrontalGyrus_male_45yrs_indiv87	brain, superior frontal gyrus, male, 45 years	87, C	superior frontal gyrus	male	age (yrs): 45			
GSM300320	EntorhinalCortex_female_47yrs_indiv88	brain, entorhinal cortex, female, 47 years	88, AA	entorhinal cortex	female	age (yrs): 47			
GSM300321	Hippocampus_female_47yrs_indiv88	brain, hippocampus, female, 47 years	88, AA	hippocampus	female	age (yrs): 47			
GSM300322	PostcentralGyrus_female_47yrs_indiv88	brain, postcentral gyrus, female, 47 years	88, AA	postcentral gyrus	female	age (yrs): 47			
GSM300323	SuperiorFrontalGyrus_female_47yrs_indiv88	brain, superior frontal gyrus, female, 47 years	88, AA	superior frontal gyrus	female	age (yrs): 47			
GSM300324	EntorhinalCortex_male_69yrs_indiv93	brain, entorhinal cortex, male, 69 years	93, C	entorhinal cortex	male	age (yrs): 69			
GSM300325	Hippocampus_male_69yrs_indiv93	brain, hippocampus, male, 69 years	93, C	hippocampus	male	age (yrs): 69			
GSM300326	PostcentralGyrus_male_69yrs_indiv93	brain, postcentral gyrus, male, 69 years	93, C	postcentral gyrus	male	age (yrs): 69			
GSM300327	SuperiorFrontalGyrus_male_69yrs_indiv93	brain, superior frontal gyrus, male, 69 years	93, C	superior frontal gyrus	male	age (yrs): 69			
GSM300328	EntorhinalCortex_female_91yrs_indiv94	brain, entorhinal cortex, female, 91 years	94, C	entorhinal cortex	female	age (yrs): 91			
GSM300329	Hippocampus_female_91yrs_indiv94	brain, hippocampus, female, 91 years	94, C	hippocampus	female	age (yrs): 91			
GSM300330	PostcentralGyrus_female_91yrs_indiv94	brain, postcentral gyrus, female, 91 years	94, C	postcentral gyrus	female	age (yrs): 91			
GSM300331	SuperiorFrontalGyrus_female_91yrs_indiv94	brain, superior frontal gyrus, female, 91 years	94, C	superior frontal gyrus	female	age (yrs): 91			
GSM300332	EntorhinalCortex_male_75yrs_indiv96	brain, entorhinal cortex, male, 75 years	96, C	entorhinal cortex	male	age (yrs): 75			
GSM300333	Hippocampus_male_75yrs_indiv96	brain, hippocampus, male, 75 years	96, C	hippocampus	male	age (yrs): 75			
GSM300335	SuperiorFrontalGyrus_male_75yrs_indiv96	brain, superior frontal gyrus, male, 75 years	96, C	superior frontal gyrus	male	age (yrs): 75			
GSM300338	EntorhinalCortex_female_82yrs_indiv98	brain, entorhinal cortex, female, 82 years	98, C	entorhinal cortex	female	age (yrs): 82			
GSM300339	Hippocampus_female_82yrs_indiv98	brain, hippocampus, female, 82 years	98, C	hippocampus	female	age (yrs): 82			
GSM300340	PostcentralGyrus_female_82yrs_indiv98	brain, postcentral gyrus, female, 82 years	98, C	postcentral gyrus	female	age (yrs): 82			
GSM300341	SuperiorFrontalGyrus_female_82yrs_indiv98	brain, superior frontal gyrus, female, 82 years	98, C	superior frontal gyrus	female	age (yrs): 82			
GSM318840	SuperiorFrontalGyrus_male_66yrs_indiv63	brain, superior frontal gyrus, male, 66 years	63, C	superior frontal gyrus	male	age (yrs): 66			
GSM350078	SuperiorFrontalGyrus_male_80yrs_indiv15	brain, superior frontal gyrus, male, 80 years	15, C	superior frontal gyrus	male	age (yrs): 80			
GSM1176196	entorhinal cortex_female_76_AD_48	entorhinal cortex_female_AD		entorhinal cortex	female	age (yrs): 76	V	3,3	3
GSM1176197	entorhinal cortex_female_86_AD_15	entorhinal cortex_female_AD		entorhinal cortex	female	age (yrs): 86	VI	4,4	9
GSM1176198	entorhinal cortex_female_82_AD_9	entorhinal cortex_female_AD		entorhinal cortex	female	age (yrs): 82	VI	3,4	0
GSM1176199	entorhinal cortex_female_85_AD_24	entorhinal cortex_female_AD		entorhinal cortex	female	age (yrs): 85	IV	3,4	26
GSM1176200	entorhinal cortex_female_90_AD_36	entorhinal cortex_female_AD		entorhinal cortex	female	age (yrs): 90	IV	3,4	17
GSM1176201	entorhinal cortex_female_90_AD_6	entorhinal cortex_female_AD		entorhinal cortex	female	age (yrs): 90	V	3,4	20
GSM1176202	entorhinal cortex_female_90_AD_75	entorhinal cortex_female_AD		entorhinal cortex	female	age (yrs): 90	III	4,4	no info

GSM1176203	entorhinal cortex_female_91_AD_11	entorhinal cortex_female_AD	entorhinal cortex	female	age (yrs): 91	V-VI	4,4	11
GSM1176204	entorhinal cortex_male_76_AD_69	entorhinal cortex_male_AD	entorhinal cortex	male	age (yrs): 76	III	4,4	no info
GSM1176205	entorhinal cortex_male_85_AD_51	entorhinal cortex_male_AD	entorhinal cortex	male	age (yrs): 85	III	3,3	22
GSM1176206	entorhinal cortex_male_85_AD_74	entorhinal cortex_male_AD	entorhinal cortex	male	age (yrs): 85	II	3,4	no info
GSM1176207	entorhinal cortex_male_86_AD_92	entorhinal cortex_male_AD	entorhinal cortex	male	age (yrs): 86	IV	3,3	19
GSM1176208	entorhinal cortex_male_87_AD_19	entorhinal cortex_male_AD	entorhinal cortex	male	age (yrs): 87	V	3,4	12
GSM1176209	entorhinal cortex_male_94_AD_5	entorhinal cortex_male_AD	entorhinal cortex	male	age (yrs): 94	VI	3,3	15
GSM1176210	entorhinal cortex_male_94_AD_90	entorhinal cortex_male_AD	entorhinal cortex	male	age (yrs): 94	IV	3,3	no info
GSM1176211	hippocampus_female_60_AD_20	hippocampus_female_AD	hippocampus	female	age (yrs): 60	VI	3,4	12
GSM1176212	hippocampus_female_74_AD_37	hippocampus_female_AD	hippocampus	female	age (yrs): 74	VI	2,4	17
GSM1176213	hippocampus_female_76_AD_48	hippocampus_female_AD	hippocampus	female	age (yrs): 76	V	3,3	3
GSM1176214	hippocampus_female_79_AD_16	hippocampus_female_AD	hippocampus	female	age (yrs): 79	VI	3,4	0
GSM1176215	hippocampus_female_86_AD_15	hippocampus_female_AD	hippocampus	female	age (yrs): 86	VI	4,4	9
GSM1176216	hippocampus_female_85_AD_24	hippocampus_female_AD	hippocampus	female	age (yrs): 85	IV	3,4	26
GSM1176217	hippocampus_female_90_AD_23	hippocampus_female_AD	hippocampus	female	age (yrs): 90	VI	3,4	12
GSM1176218	hippocampus_female_90_AD_36	hippocampus_female_AD	hippocampus	female	age (yrs): 90	IV	3,4	17
GSM1176219	hippocampus_female_90_AD_75	hippocampus_female_AD	hippocampus	female	age (yrs): 90	III	4,4	no info
GSM1176220	hippocampus_female_91_AD_11	hippocampus_female_AD	hippocampus	female	age (yrs): 91	V-VI	4,4	11
GSM1176221	hippocampus_male_76_AD_33	hippocampus_male_AD	hippocampus	male	age (yrs): 76	V	4,4	0
GSM1176222	hippocampus_male_76_AD_69	hippocampus_male_AD	hippocampus	male	age (yrs): 76	III	4,4	no info
GSM1176223	hippocampus_male_79_AD_7	hippocampus_male_AD	hippocampus	male	age (yrs): 79	VI	3,4	6
GSM1176224	hippocampus_male_80_AD_43	hippocampus_male_AD	hippocampus	male	age (yrs): 80	no info	3,3	23
GSM1176225	hippocampus_male_85_AD_51	hippocampus_male_AD	hippocampus	male	age (yrs): 85	III	3,3	22
GSM1176226	hippocampus_male_86_AD_92	hippocampus_male_AD	hippocampus	male	age (yrs): 86	IV	3,3	19
GSM1176227	hippocampus_male_87_AD_19	hippocampus_male_AD	hippocampus	male	age (yrs): 87	V	3,4	12
GSM1176228	hippocampus_male_94_AD_5	hippocampus_male_AD	hippocampus	male	age (yrs): 94	VI	3,3	15
GSM1176229	hippocampus_male_94_AD_90	hippocampus_male_AD	hippocampus	male	age (yrs): 94	IV	3,3	no info
GSM1176230	post-central gyrus_female_60_AD_20	post-central gyrus_female_AD	post-central gyrus	female	age (yrs): 60	VI	3,4	12
GSM1176231	post-central gyrus_female_74_AD_37	post-central gyrus_female_AD	post-central gyrus	female	age (yrs): 74	VI	2,4	17
GSM1176232	post-central gyrus_female_76_AD_48	post-central gyrus_female_AD	post-central gyrus	female	age (yrs): 76	V	3,3	3
GSM1176233	post-central gyrus_female_86_AD_15	post-central gyrus_female_AD	post-central gyrus	female	age (yrs): 86	VI	4,4	9
GSM1176234	post-central gyrus_female_82_AD_13	post-central gyrus_female_AD	post-central gyrus	female	age (yrs): 82	VI	3,4	4
GSM1176235	post-central gyrus_female_82_AD_9	post-central gyrus_female_AD	post-central gyrus	female	age (yrs): 82	VI	3,4	0
GSM1176236	post-central gyrus_female_85_AD_24	post-central gyrus_female_AD	post-central gyrus	female	age (yrs): 85	IV	3,4	26
GSM1176237	post-central gyrus_female_85_AD_57	post-central gyrus_female_AD	post-central gyrus	female	age (yrs): 85	V	3,3	no info
GSM1176238	post-central gyrus_female_90_AD_23	post-central gyrus_female_AD	post-central gyrus	female	age (yrs): 90	VI	3,4	12
GSM1176239	post-central gyrus_female_90_AD_36	post-central gyrus_female_AD	post-central gyrus	female	age (yrs): 90	IV	3,4	17
GSM1176240	post-central gyrus_female_90_AD_6	post-central gyrus_female_AD	post-central gyrus	female	age (yrs): 90	V	3,4	20
GSM1176241	post-central gyrus_female_90_AD_75	post-central gyrus_female_AD	post-central gyrus	female	age (yrs): 90	III	4,4	no info
GSM1176242	post-central gyrus_female_91_AD_11	post-central gyrus_female_AD	post-central gyrus	female	age (yrs): 91	V-VI	4,4	11
GSM1176243	post-central gyrus_female_94_AD_70	post-central gyrus_female_AD	post-central gyrus	female	age (yrs): 94	I	3,3	no info
GSM1176244	post-central gyrus_female_95_AD_64	post-central gyrus_female_AD	post-central gyrus	female	age (yrs): 95	no info	3,3	no info
GSM1176245	post-central gyrus_male_76_AD_33	post-central gyrus_male_AD	post-central gyrus	male	age (yrs): 76	V	4,4	0
GSM1176246	post-central gyrus_male_76_AD_69	post-central gyrus_male_AD	post-central gyrus	male	age (yrs): 76	III	4,4	no info
GSM1176247	post-central gyrus_male_79_AD_7	post-central gyrus_male_AD	post-central gyrus	male	age (yrs): 79	VI	3,4	6
GSM1176248	post-central gyrus_male_85_AD_51	post-central gyrus_male_AD	post-central gyrus	male	age (yrs): 85	III	3,3	22
GSM1176249	post-central gyrus_male_85_AD_74	post-central gyrus_male_AD	post-central gyrus	male	age (yrs): 85	II	3,4	no info
GSM1176250	post-central gyrus_male_86_AD_92	post-central gyrus_male_AD	post-central gyrus	male	age (yrs): 86	IV	3,3	19
GSM1176251	post-central gyrus_male_87_AD_19	post-central gyrus_male_AD	post-central gyrus	male	age (yrs): 87	V	3,4	12
GSM1176252	post-central gyrus_male_94_AD_44	post-central gyrus_male_AD	post-central gyrus	male	age (yrs): 94	IV	3,4	18
GSM1176253	post-central gyrus_male_94_AD_5	post-central gyrus_male_AD	post-central gyrus	male	age (yrs): 94	VI	3,3	15
GSM1176254	post-central gyrus_male_94_AD_90	post-central gyrus_male_AD	post-central gyrus	male	age (yrs): 94	IV	3,3	no info
GSM1176255	superior frontal gyrus_female_74_AD_37	superior frontal gyrus_female_AD	superior frontal gyrus	female	age (yrs): 74	VI	2,4	17
GSM1176256	superior frontal gyrus_female_76_AD_48	superior frontal gyrus_female_AD	superior frontal gyrus	female	age (yrs): 76	V	3,3	3
GSM1176257	superior frontal gyrus_female_86_AD_15	superior frontal gyrus_female_AD	superior frontal gyrus	female	age (yrs): 86	VI	4,4	9
GSM1176258	superior frontal gyrus_female_82_AD_9	superior frontal gyrus_female_AD	superior frontal gyrus	female	age (yrs): 82	VI	3,4	0
GSM1176259	superior frontal gyrus_female_85_AD_24	superior frontal gyrus_female_AD	superior frontal gyrus	female	age (yrs): 85	IV	3,4	26
GSM1176260	superior frontal gyrus_female_85_AD_57	superior frontal gyrus_female_AD	superior frontal gyrus	female	age (yrs): 85	V	3,3	no info
GSM1176261	superior frontal gyrus_female_88_AD_91	superior frontal gyrus_female_AD	superior frontal gyrus	female	age (yrs): 88	IV	3,4	27
GSM1176262	superior frontal gyrus_female_90_AD_23	superior frontal gyrus_female_AD	superior frontal gyrus	female	age (yrs): 90	VI	3,4	12
GSM1176263	superior frontal gyrus_female_90_AD_36	superior frontal gyrus_female_AD	superior frontal gyrus	female	age (yrs): 90	IV	3,4	17

GSM1176264	superior frontal gyrus_female_90_AD_6	superior frontal gyrus_female_AD	superior frontal gyrus	female	age (yrs): 90	V	3,4	20
GSM1176265	superior frontal gyrus_female_90_AD_75	superior frontal gyrus_female_AD	superior frontal gyrus	female	age (yrs): 90	III	4,4	no info
GSM1176266	superior frontal gyrus_female_91_AD_11	superior frontal gyrus_female_AD	superior frontal gyrus	female	age (yrs): 91	V-VI	4,4	11
GSM1176267	superior frontal gyrus_female_94_AD_70	superior frontal gyrus_female_AD	superior frontal gyrus	female	age (yrs): 94	I	3,3	no info
GSM1176268	superior frontal gyrus_female_95_AD_64	superior frontal gyrus_female_AD	superior frontal gyrus	female	age (yrs): 95	no info	3,3	no info
GSM1176269	superior frontal gyrus_male_76_AD_33	superior frontal gyrus_male_AD	superior frontal gyrus	male	age (yrs): 76	V	4,4	0
GSM1176270	superior frontal gyrus_male_85_AD_51	superior frontal gyrus_male_AD	superior frontal gyrus	male	age (yrs): 85	III	3,3	22
GSM1176271	superior frontal gyrus_male_85_AD_74	superior frontal gyrus_male_AD	superior frontal gyrus	male	age (yrs): 85	II	3,4	no info
GSM1176272	superior frontal gyrus_male_86_AD_92	superior frontal gyrus_male_AD	superior frontal gyrus	male	age (yrs): 86	IV	3,3	19
GSM1176273	superior frontal gyrus_male_94_AD_44	superior frontal gyrus_male_AD	superior frontal gyrus	male	age (yrs): 94	IV	3,4	18
GSM1176274	superior frontal gyrus_male_94_AD_5	superior frontal gyrus_male_AD	superior frontal gyrus	male	age (yrs): 94	VI	3,3	15
GSM1176275	superior frontal gyrus_male_94_AD_90	superior frontal gyrus_male_AD	superior frontal gyrus	male	age (yrs): 94	IV	3,3	no info
