# Supplementary material for: Integrated analysis and identification of hub genes as novel biomarkers for Alzheimer’s disease
Source: Front Aging Neurosci. 2022 Aug 30;14:901972. doi: 10.3389/fnagi.2022.901972 (PMC9468260; doi:10.3389/fnagi.2022.901972)
Supplement: Supplementary file 2 [file Data_Sheet_2.pdf]

GSE5281

Group	Accession	Title	Organ Region	Cell Type	Sex	Age
normal	GSM119615	EC control 1	Entorhinal Cortex	layer III neurons	male	63
normal	GSM119616	EC control 2	Entorhinal Cortex	layer III neurons	male	85
normal	GSM119617	EC control 3	Entorhinal Cortex	layer III neurons	male	80
normal	GSM119618	EC control 4	Entorhinal Cortex	layer III neurons	male	80
normal	GSM119619	EC control 5	Entorhinal Cortex	layer III neurons	female	102
normal	GSM119620	EC control 6	Entorhinal Cortex	layer III neurons	male	79
normal	GSM119621	EC control 7	Entorhinal Cortex	layer III neurons	male	76
normal	GSM119622	EC control 8	Entorhinal Cortex	layer III neurons	male	83
normal	GSM119623	EC control 9	Entorhinal Cortex	layer III neurons	male	79
normal	GSM119624	EC control 10	Entorhinal Cortex	layer III neurons	female	88
normal	GSM119625	EC control 11	Entorhinal Cortex	layer III neurons	female	82
normal	GSM119626	EC control 12	Entorhinal Cortex	layer III neurons	male	69
normal	GSM119627	EC control 13	Entorhinal Cortex	layer III neurons	male	78
normal	GSM119628	HIP control 1	hippocampus	layer III neurons	male	85
normal	GSM119629	HIP control 2	hippocampus	layer III neurons	male	80
normal	GSM119630	HIP control 3	hippocampus	layer III neurons	male	80
normal	GSM119631	HIP control 4	hippocampus	layer III neurons	female	102
normal	GSM119632	HIP control 5	hippocampus	layer III neurons	male	63
normal	GSM119633	HIP control 6	hippocampus	layer III neurons	male	79
normal	GSM119634	HIP control 7	hippocampus	layer III neurons	male	76
normal	GSM119635	HIP control 8	hippocampus	layer III neurons	male	83
normal	GSM119636	HIP control 9	hippocampus	layer III neurons	male	79
normal	GSM119637	HIP control 10	hippocampus	layer III neurons	female	88
normal	GSM119638	HIP control 11	hippocampus	layer III neurons	female	73
normal	GSM119639	HIP control 12	hippocampus	layer III neurons	male	69
normal	GSM119640	HIP control 13	hippocampus	layer III neurons	male	78
normal	GSM119641	MTG control 1	Medial Temporal Gyrus	layer III neurons	male	85
normal	GSM119642	MTG control 2	Medial Temporal Gyrus	layer III neurons	male	80
normal	GSM119643	MTG control 3	Medial Temporal Gyrus	layer III neurons	female	102
normal	GSM119644	MTG control 4	Medial Temporal Gyrus	layer III neurons	male	63
normal	GSM119645	MTG control 5	Medial Temporal Gyrus	layer III neurons	male	79
normal	GSM119646	MTG control 6	Medial Temporal Gyrus	layer III neurons	male	83
normal	GSM119647	MTG control 7	Medial Temporal Gyrus	layer III neurons	male	79
normal	GSM119648	MTG control 8	Medial Temporal Gyrus	layer III neurons	female	88
normal	GSM119649	MTG control 9	Medial Temporal Gyrus	layer III neurons	female	82
normal	GSM119650	MTG control 10	Medial Temporal Gyrus	layer III neurons	female	73

normal	GSM119651	MTG control 11	Medial Temporal Gyrus	layer III neurons	male	69
normal	GSM119652	MTG control 12	Medial Temporal Gyrus	layer III neurons	male	78
normal	GSM119653	PC control 1	Posterior Cingulate	layer III neurons	male	85
normal	GSM119654	PC control 2	Posterior Cingulate	layer III neurons	male	80
normal	GSM119655	PC control 3	Posterior Cingulate	layer III neurons	female	102
normal	GSM119656	PC control 4	Posterior Cingulate	layer III neurons	male	63
normal	GSM119657	PC control 5	Posterior Cingulate	layer III neurons	male	79
normal	GSM119658	PC control 6	Posterior Singulate	layer III neurons	male	76
normal	GSM119659	PC control 7	Posterior cingulate	layer III neurons	male	83
normal	GSM119660	PC control 8	Posterior cingulate	layer III neurons	male	79
normal	GSM119661	PC control 9	Posterior cingulate	layer III neurons	female	88
normal	GSM119662	PC control 10	Posterior cingulate	layer III neurons	female	82
normal	GSM119663	PC control 11	Posterior cingulate	layer III neurons	female	73
normal	GSM119664	PC control 12	Posterior cingulate	layer III neurons	male	69
normal	GSM119665	PC control 13	Posterior cingulate	layer III neurons	male	78
normal	GSM119666	SFG control 1	Superior Frontal Gyrus	layer III neurons	male	79
normal	GSM119667	SFG control 2	Superior Frontal Gyrus	layer III neurons	female	88
normal	GSM119668	SFG control 3	Superior Frontal Gyrus	layer III neurons	female	82
normal	GSM119669	SFG control 4	Superior Frontal Gyrus	layer III neurons	female	73
normal	GSM119670	SFG control 5	Superior Frontal Gyrus	layer III neurons	female	102
normal	GSM119671	SFG control 6	Superior Frontal Gyrus	layer III neurons	male	63
normal	GSM119672	SFG control 7	Superior Frontal Gyrus	layer III neurons	male	79
normal	GSM119673	SFG control 8	Superior Frontal Gyrus	layer III neurons	male	76
normal	GSM119674	SFG control 9	Superior Frontal Gyrus	layer III neurons	male	83
normal	GSM119675	SFG control 10	Superior Frontal Gyrus	layer III neurons	male	69
normal	GSM119676	SFG control 11	Superior Frontal Gyrus	layer III neurons	male	78
normal	GSM119677	VCX control 1	Primary Visual Cortex	layer III neurons	male	85
normal	GSM119678	VCX control 2	Primary Visual Cortex	layer III neurons	male	80
normal	GSM119679	VCX control 3	Primary Visual Cortex	layer III neurons	male	63
normal	GSM119680	VCX control 4	Primary Visual Cortex	layer III neurons	male	79
normal	GSM119681	VCX control 5	Primary Visual Cortex	layer III neurons	male	76
normal	GSM119682	VCX control 6	Primary Visual Cortex	layer III neurons	male	83
normal	GSM119683	VCX control 7	Primary Visual Cortex	layer III neurons	male	79
normal	GSM119684	VCX control 8	Primary Visual Cortex	layer III neurons	female	88
normal	GSM119685	VCX control 9	Primary Visual Cortex	layer III neurons	female	82
normal	GSM119686	VCX control 10	Primary Visual Cortex	layer III neurons	female	73
normal	GSM119687	VCX control 11	Primary Visual Cortex	layer III neurons	male	69
normal	GSM119688	VCX control 12	Primary Visual Cortex	layer III neurons	male	78
Alzheimer's Disease	GSM238763	EC_affected_1	Entorhinal Cortex	pyramidal neuron	female	82
Alzheimer's Disease	GSM238790	EC_affected_2	Entorhinal Cortex	pyramidal neuron	female	86

Alzheimer's Disease	GSM238791	EC_affected_3	Entorhinal Cortex	pyramidal neuron	female	93
Alzheimer's Disease	GSM238792	EC_affected_4	Entorhinal Cortex	pyramidal neuron	male	84
Alzheimer's Disease	GSM238793	EC_affected_5	Entorhinal Cortex	pyramidal neuron	female	79
Alzheimer's Disease	GSM238794	EC_affected_6	Entorhinal Cortex	pyramidal neuron	female	78
Alzheimer's Disease	GSM238795	EC_affected_7	Entorhinal Cortex	pyramidal neuron	female	91
Alzheimer's Disease	GSM238796	EC_affected_8	Entorhinal Cortex	pyramidal neuron	male	86
Alzheimer's Disease	GSM238797	EC_affected_9	Entorhinal Cortex	pyramidal neuron	male	97
Alzheimer's Disease	GSM238798	EC_affected_10	Entorhinal Cortex	pyramidal neuron	male	80
Alzheimer's Disease	GSM238799	HIP_affected_1	hippocampus	pyramidal neuron	male	72
Alzheimer's Disease	GSM238800	HIP_affected_2	hippocampus	pyramidal neuron	female	73
Alzheimer's Disease	GSM238801	HIP_affected_3	hippocampus	pyramidal neuron	male	75
Alzheimer's Disease	GSM238802	HIP_affected_4	hippocampus	pyramidal neuron	female	70
Alzheimer's Disease	GSM238803	HIP_affected_5	hippocampus	pyramidal neuron	female	85
Alzheimer's Disease	GSM238804	HIP_affected_6	hippocampus	pyramidal neuron	female	77
Alzheimer's Disease	GSM238805	HIP_affected_7	hippocampus	pyramidal neuron	male	79
Alzheimer's Disease	GSM238806	HIP_affected_8	hippocampus	pyramidal neuron	male	88
Alzheimer's Disease	GSM238807	HIP_affected_9	hippocampus	pyramidal neuron	male	81
Alzheimer's Disease	GSM238808	HIP_affected_10	hippocampus	layer III neurons	male	78
Alzheimer's Disease	GSM238809	MTG_affected_1	Medial Temporal Gyrus	pyramidal neuron	female	73
Alzheimer's Disease	GSM238810	MTG_affected_2	Medial Temporal Gyrus	pyramidal neuron	male	81
Alzheimer's Disease	GSM238811	MTG_affected_3	Medial Temporal Gyrus	pyramidal neuron	male	72
Alzheimer's Disease	GSM238812	MTG_affected_4	Medial Temporal Gyrus	pyramidal neuron	male	75
Alzheimer's Disease	GSM238813	MTG_affected_5	Medial Temporal Gyrus	pyramidal neuron	male	78
Alzheimer's Disease	GSM238815	MTG_affected_6	Medial Temporal Gyrus	pyramidal neuron	male	75
Alzheimer's Disease	GSM238816	MTG_affected_7	Medial Temporal Gyrus	pyramidal neuron	male	87
Alzheimer's Disease	GSM238817	MTG_affected_8	Medial Temporal Gyrus	pyramidal neuron	female	>90
Alzheimer's Disease	GSM238818	MTG_affected_9	Medial Temporal Gyrus	pyramidal neuron	male	68
Alzheimer's Disease	GSM238819	MTG_affected_10	Medial Temporal Gyrus	pyramidal neuron	female	81
Alzheimer's Disease	GSM238820	MTG_affected_11	Medial Temporal Gyrus	pyramidal neuron	female	85
Alzheimer's Disease	GSM238821	MTG_affected_12	Medial Temporal Gyrus	pyramidal neuron	male	79
Alzheimer's Disease	GSM238822	MTG_affected_13	Medial Temporal Gyrus	layer III neurons	female	82
Alzheimer's Disease	GSM238823	MTG_affected_14	Medial Temporal Gyrus	layer III neurons	male	88
Alzheimer's Disease	GSM238824	MTG_affected_15	Medial Temporal Gyrus	layer III neurons	male	72
Alzheimer's Disease	GSM238825	MTG_affected_16	Medial Temporal Gyrus	layer III neurons	female	80
Alzheimer's Disease	GSM238826	PC_affected_1	Posterior Cingulate	pyramidal neuron	female	73
Alzheimer's Disease	GSM238827	PC_affected_2	Posterior Singulate	pyramidal neuron	male	81
Alzheimer's Disease	GSM238834	PC_affected_3	Posterior Singulate	pyramidal neuron	male	78
Alzheimer's Disease	GSM238835	PC_affected_4	Posterior Singulate	pyramidal neuron	male	75
Alzheimer's Disease	GSM238837	PC_affected_5	Posterior Singulate	pyramidal neuron	male	68
Alzheimer's Disease	GSM238838	PC_affected_6	Posterior Singulate	pyramidal neuron	female	70.8

Alzheimer's Disease	GSM238839	PC_affected_7	Posterior Singulate	pyramidal neuron	female	85
Alzheimer's Disease	GSM238840	PC_affected_8	Posterior Singulate	pyramidal neuron	male	79
Alzheimer's Disease	GSM238841	PC_affected_9	Posterior Singulate	pyramidal neuron	male	88
Alzheimer's Disease	GSM238842	SFG_affected_1	Superior Frontal Gyrus	pyramidal neuron	female	73
Alzheimer's Disease	GSM238843	SFG_affected_2	Superior Frontal Gyrus	pyramidal neuron	male	81
Alzheimer's Disease	GSM238844	SFG_affected_3	Superior Frontal Gyrus	pyramidal neuron	male	72
Alzheimer's Disease	GSM238845	SFG_affected_4	Superior Frontal Gyrus	pyramidal neuron	male	75
Alzheimer's Disease	GSM238846	SFG_affected_5	Superior Frontal Gyrus	pyramidal neuron	male	78
Alzheimer's Disease	GSM238847	SFG_affected_6	Superior Frontal Gyrus	pyramidal neuron	male	75
Alzheimer's Disease	GSM238848	SFG_affected_7	Superior Frontal Gyrus	pyramidal neuron	male	87
Alzheimer's Disease	GSM238851	SFG_affected_8	Superior Frontal Gyrus	pyramidal neuron	female	95
Alzheimer's Disease	GSM238854	SFG_affected_9	Superior Frontal Gyrus	pyramidal neuron	male	68
Alzheimer's Disease	GSM238855	SFG_affected_10	Superior Frontal Gyrus	pyramidal neuron	female	95
Alzheimer's Disease	GSM238856	SFG_affected_11	Superior Frontal Gyrus	pyramidal neuron	female	70.8
Alzheimer's Disease	GSM238857	SFG_affected_12	Superior Frontal Gyrus	pyramidal neuron	female	85
Alzheimer's Disease	GSM238858	SFG_affected_13	Superior Frontal Gyrus	pyramidal neuron	female	83
Alzheimer's Disease	GSM238860	SFG_affected_14	Superior Frontal Gyrus	pyramidal neuron	female	77
Alzheimer's Disease	GSM238861	SFG_affected_15	Superior Frontal Gyrus	pyramidal neuron	female	83
Alzheimer's Disease	GSM238862	SFG_affected_16	Superior Frontal Gyrus	pyramidal neuron	male	68
Alzheimer's Disease	GSM238863	SFG_affected_17	Superior Frontal Gyrus	pyramidal neuron	male	79
Alzheimer's Disease	GSM238864	SFG_affected_18	Superior Frontal Gyrus	pyramidal neuron	female	82
Alzheimer's Disease	GSM238865	SFG_affected_19	Superior Frontal Gyrus	pyramidal neuron	male	80
Alzheimer's Disease	GSM238867	SFG_affected_20	Superior Frontal Gyrus	pyramidal neuron	male	88
Alzheimer's Disease	GSM238868	SFG_affected_21	Superior Frontal Gyrus	pyramidal neuron	male	74
Alzheimer's Disease	GSM238870	SFG_affected_22	Superior Frontal Gyrus	pyramidal neuron	male	72
Alzheimer's Disease	GSM238871	SFG_affected_23	Superior Frontal Gyrus	layer III neurons	female	80
Alzheimer's Disease	GSM238872	VCX_affected_1	Primary Visual Cortex	pyramidal neuron	female	73
Alzheimer's Disease	GSM238873	VCX_affected_2	Primary Visual Cortex	pyramidal neuron	male	81
Alzheimer's Disease	GSM238874	VCX_affected_3	Primary Visual Cortex	pyramidal neuron	male	75
Alzheimer's Disease	GSM238875	VCX_affected_4	Primary Visual Cortex	pyramidal neuron	male	78
Alzheimer's Disease	GSM238877	VCX_affected_5	Primary Visual Cortex	pyramidal neuron	male	75
Alzheimer's Disease	GSM238941	VCX_affected_6	Primary Visual Cortex	pyramidal neuron	male	87
Alzheimer's Disease	GSM238942	VCX_affected_7	Primary Visual Cortex	pyramidal neuron	female	95
Alzheimer's Disease	GSM238943	VCX_affected_8	Primary Visual Cortex	pyramidal neuron	male	68
Alzheimer's Disease	GSM238944	VCX_affected_9	Primary Visual Cortex	pyramidal neuron	female	>90
Alzheimer's Disease	GSM238945	VCX_affected_10	Primary Visual Cortex	pyramidal neuron	female	81.3
Alzheimer's Disease	GSM238946	VCX_affected_11	Primary Visual Cortex	pyramidal neuron	female	81
Alzheimer's Disease	GSM238947	VCX_affected_12	Primary Visual Cortex	pyramidal neuron	female	85
Alzheimer's Disease	GSM238948	VCX_affected_13	Primary Visual Cortex	pyramidal neuron	male	79
Alzheimer's Disease	GSM238949	VCX_affected_14	Primary Visual Cortex	pyramidal neuron	male	79

Alzheimer's Disease	GSM238951	VCX_affected_15	Primary Visual Cortex	pyramidal neuron	female	82
Alzheimer's Disease	GSM238952	VCX_affected_16	Primary Visual Cortex	pyramidal neuron	male	88
Alzheimer's Disease	GSM238953	VCX_affected_17	Primary Visual Cortex	pyramidal neuron	male	74
Alzheimer's Disease	GSM238955	VCX_affected_18	Primary Visual Cortex	layer III neurons	male	72
Alzheimer's Disease	GSM238963	VCX_affected_19	Primary Visual Cortex	layer III neurons	female	80

GSE28146

Group	Accession	Title	Sex	Age
normal	GSM697308	Control 976	Male	85
normal	GSM697309	Control 1003	Male	80
normal	GSM697310	Control 1008	Female	92
normal	GSM697311	Control 1012	Male	80
normal	GSM697312	Control 1015	Male	75
normal	GSM697313	Control 1018	Female	97
normal	GSM697314	Control 1030	Male	95
normal	GSM697315	Control 1040	Male	87
Alzheimer's Disease	GSM697316	Incipient 715	Female	101
Alzheimer's Disease	GSM697317	Incipient 720	Female	95
Alzheimer's Disease	GSM697318	Incipient 994	Female	83
Alzheimer's Disease	GSM697319	Incipient 1019	Male	88
Alzheimer's Disease	GSM697320	Incipient 1029	Female	91
Alzheimer's Disease	GSM697321	Incipient 1034	Male	88
Alzheimer's Disease	GSM697322	Incipient 1043	Female	97
Alzheimer's Disease	GSM697323	Moderate 826	Female	85
Alzheimer's Disease	GSM697324	Moderate 832	Female	89
Alzheimer's Disease	GSM697325	Moderate 856	Female	83
Alzheimer's Disease	GSM697326	Moderate 965	Female	82
Alzheimer's Disease	GSM697327	Moderate 1020	Female	79
Alzheimer's Disease	GSM697328	Moderate 1025	Male	81
Alzheimer's Disease	GSM697329	Moderate 1031	Female	86
Alzheimer's Disease	GSM697330	Moderate 1037	Male	82
Alzheimer's Disease	GSM697331	Severe 701	Male	85
Alzheimer's Disease	GSM697332	Severe 723	Female	65
Alzheimer's Disease	GSM697333	Severe 807	Male	93
Alzheimer's Disease	GSM697334	Severe 819	Female	79
Alzheimer's Disease	GSM697335	Severe 867	Female	94
Alzheimer's Disease	GSM697336	Severe 872	Female	79
Alzheimer's Disease	GSM697337	Severe 1036	Female	93

GSE48350

Group	Accession	Title	Organ Region	Gender	Age
normal	GSM300338	EntorhinalCortex_female_82yrs_indiv98	entorhinal cortex	female	82
normal	GSM300339	Hippocampus_female_82yrs_indiv98	hippocampus	female	82
normal	GSM300340	PostcentralGyrus_female_82yrs_indiv98	postcentral gyrus	female	82
normal	GSM300341	SuperiorFrontalGyrus_female_82yrs_indiv98	superior frontal gyrus	female	82
normal	GSM300332	EntorhinalCortex_male_75yrs_indiv96	entorhinal cortex	male	75
normal	GSM300333	Hippocampus_male_75yrs_indiv96	hippocampus	male	75
normal	GSM300335	SuperiorFrontalGyrus_male_75yrs_indiv96	superior frontal gyrus	male	75
normal	GSM300328	EntorhinalCortex_female_91yrs_indiv94	entorhinal cortex	female	91
normal	GSM300329	Hippocampus_female_91yrs_indiv94	hippocampus	female	91
normal	GSM300330	PostcentralGyrus_female_91yrs_indiv94	postcentral gyrus	female	91
normal	GSM300331	SuperiorFrontalGyrus_female_91yrs_indiv94	superior frontal gyrus	female	91
normal	GSM300324	EntorhinalCortex_male_69yrs_indiv93	entorhinal cortex	male	69
normal	GSM300325	Hippocampus_male_69yrs_indiv93	hippocampus	male	69
normal	GSM300326	PostcentralGyrus_male_69yrs_indiv93	postcentral gyrus	male	69
normal	GSM300327	SuperiorFrontalGyrus_male_69yrs_indiv93	superior frontal gyrus	male	69
normal	GSM300320	EntorhinalCortex_female_47yrs_indiv88	entorhinal cortex	female	47
normal	GSM300321	Hippocampus_female_47yrs_indiv88	hippocampus	female	47
normal	GSM300322	PostcentralGyrus_female_47yrs_indiv88	postcentral gyrus	female	47
normal	GSM300323	SuperiorFrontalGyrus_female_47yrs_indiv88	superior frontal gyrus	female	47
normal	GSM300316	EntorhinalCortex_male_45yrs_indiv87	entorhinal cortex	male	45
normal	GSM300317	Hippocampus_male_45yrs_indiv87	hippocampus	male	45
normal	GSM300318	PostcentralGyrus_male_45yrs_indiv87	postcentral gyrus	male	45
normal	GSM300319	SuperiorFrontalGyrus_male_45yrs_indiv87	superior frontal gyrus	male	45
normal	GSM300312	EntorhinalCortex_male_42yrs_indiv86	entorhinal cortex	male	42
normal	GSM300313	Hippocampus_male_42yrs_indiv86	hippocampus	male	42
normal	GSM300314	PostcentralGyrus_male_42yrs_indiv86	postcentral gyrus	male	42
normal	GSM300315	SuperiorFrontalGyrus_male_42yrs_indiv86	superior frontal gyrus	male	42
normal	GSM300308	EntorhinalCortex_male_22yrs_indiv85	entorhinal cortex	male	22
normal	GSM300309	Hippocampus_male_22yrs_indiv85	hippocampus	male	22
normal	GSM300310	PostcentralGyrus_male_22yrs_indiv85	postcentral gyrus	male	22
normal	GSM300311	SuperiorFrontalGyrus_male_22yrs_indiv85	superior frontal gyrus	male	22
normal	GSM300304	EntorhinalCortex_male_33yrs_indiv84	entorhinal cortex	male	33
normal	GSM300305	Hippocampus_male_33yrs_indiv84	hippocampus	male	33
normal	GSM300306	PostcentralGyrus_male_33yrs_indiv84	postcentral gyrus	male	33
normal	GSM300307	SuperiorFrontalGyrus_male_33yrs_indiv84	superior frontal gyrus	male	33
normal	GSM300300	EntorhinalCortex_male_20yrs_indiv83	entorhinal cortex	male	20

normal	GSM300301	Hippocampus_male_20yrs_indiv83	hippocampus	male	20
normal	GSM300302	PostcentralGyrus_male_20yrs_indiv83	postcentral gyrus	male	20
normal	GSM300303	SuperiorFrontalGyrus_male_20yrs_indiv83	superior frontal gyrus	male	20
normal	GSM300297	EntorhinalCortex_female_30yrs_indiv82	entorhinal cortex	female	30
normal	GSM300298	Hippocampus_female_30yrs_indiv82	hippocampus	female	30
normal	GSM300299	SuperiorFrontalGyrus_female_30yrs_indiv82	superior frontal gyrus	female	30
normal	GSM300293	EntorhinalCortex_female_48yrs_indiv81	entorhinal cortex	female	48
normal	GSM300294	Hippocampus_female_48yrs_indiv81	hippocampus	female	48
normal	GSM300295	PostcentralGyrus_female_48yrs_indiv81	postcentral gyrus	female	48
normal	GSM300296	SuperiorFrontalGyrus_female_48yrs_indiv81	superior frontal gyrus	female	48
normal	GSM300289	EntorhinalCortex_female_44yrs_indiv80	entorhinal cortex	female	44
normal	GSM300290	Hippocampus_female_44yrs_indiv80	hippocampus	female	44
normal	GSM300291	PostcentralGyrus_female_44yrs_indiv80	postcentral gyrus	female	44
normal	GSM300292	SuperiorFrontalGyrus_female_44yrs_indiv80	superior frontal gyrus	female	44
normal	GSM300285	EntorhinalCortex_male_69yrs_indiv8	entorhinal cortex	male	69
normal	GSM300286	Hippocampus_male_69yrs_indiv8	hippocampus	male	69
normal	GSM300287	PostcentralGyrus_male_69yrs_indiv8	postcentral gyrus	male	69
normal	GSM300288	SuperiorFrontalGyrus_male_69yrs_indiv8	superior frontal gyrus	male	69
normal	GSM300283	PostcentralGyrus_female_36yrs_indiv79	postcentral gyrus	female	36
normal	GSM300284	SuperiorFrontalGyrus_female_36yrs_indiv79	superior frontal gyrus	female	36
normal	GSM300279	EntorhinalCortex_male_20yrs_indiv78	entorhinal cortex	male	20
normal	GSM300280	Hippocampus_male_20yrs_indiv78	hippocampus	male	20
normal	GSM300281	PostcentralGyrus_male_20yrs_indiv78	postcentral gyrus	male	20
normal	GSM300282	SuperiorFrontalGyrus_male_20yrs_indiv78	superior frontal gyrus	male	20
normal	GSM300275	EntorhinalCortex_male_20yrs_indiv77	entorhinal cortex	male	20
normal	GSM300276	Hippocampus_male_20yrs_indiv77	hippocampus	male	20
normal	GSM300277	PostcentralGyrus_male_20yrs_indiv77	postcentral gyrus	male	20
normal	GSM300278	SuperiorFrontalGyrus_male_20yrs_indiv77	superior frontal gyrus	male	20
normal	GSM300271	EntorhinalCortex_female_26yrs_indiv76	entorhinal cortex	female	26
normal	GSM300272	Hippocampus_female_26yrs_indiv76	hippocampus	female	26
normal	GSM300273	PostcentralGyrus_female_26yrs_indiv76	postcentral gyrus	female	26
normal	GSM300274	SuperiorFrontalGyrus_female_26yrs_indiv76	superior frontal gyrus	female	26
normal	GSM300267	EntorhinalCortex_male_86yrs_indiv73	entorhinal cortex	male	86
normal	GSM300268	Hippocampus_male_86yrs_indiv73	hippocampus	male	86
normal	GSM300269	PostcentralGyrus_male_86yrs_indiv73	postcentral gyrus	male	86
normal	GSM300270	SuperiorFrontalGyrus_male_86yrs_indiv73	superior frontal gyrus	male	86
normal	GSM300265	PostcentralGyrus_male_75yrs_indiv72	postcentral gyrus	male	75
normal	GSM300266	SuperiorFrontalGyrus_male_75yrs_indiv72	superior frontal gyrus	male	75
normal	GSM300261	EntorhinalCortex_male_52yrs_indiv71	entorhinal cortex	male	52
normal	GSM300262	Hippocampus_male_52yrs_indiv71	hippocampus	male	52

normal	GSM300263	PostcentralGyrus_male_52yrs_indiv71	postcentral gyrus	male	52
normal	GSM300264	SuperiorFrontalGyrus_male_52yrs_indiv71	superior frontal gyrus	male	52
normal	GSM300258	EntorhinalCortex_male_40yrs_indiv68	entorhinal cortex	male	40
normal	GSM300259	PostcentralGyrus_male_40yrs_indiv68	postcentral gyrus	male	40
normal	GSM300260	SuperiorFrontalGyrus_male_40yrs_indiv68	superior frontal gyrus	male	40
normal	GSM300255	Hippocampus_male_69yrs_indiv67	hippocampus	male	69
normal	GSM300256	PostcentralGyrus_male_69yrs_indiv67	postcentral gyrus	male	69
normal	GSM300252	EntorhinalCortex_male_21yrs_indiv66	entorhinal cortex	male	21
normal	GSM300253	PostcentralGyrus_male_21yrs_indiv66	postcentral gyrus	male	21
normal	GSM300254	SuperiorFrontalGyrus_male_21yrs_indiv66	superior frontal gyrus	male	21
normal	GSM318840	SuperiorFrontalGyrus_male_66yrs_indiv63	superior frontal gyrus	male	66
normal	GSM300251	SuperiorFrontalGyrus_female_91yrs_indiv59	superior frontal gyrus	female	91
normal	GSM300248	EntorhinalCortex_female_85yrs_indiv54	entorhinal cortex	female	85
normal	GSM300249	PostcentralGyrus_female_85yrs_indiv54	postcentral gyrus	female	85
normal	GSM300250	SuperiorFrontalGyrus_female_85yrs_indiv54	superior frontal gyrus	female	85
normal	GSM300246	PostcentralGyrus_male_70yrs_indiv53	postcentral gyrus	male	70
normal	GSM300247	SuperiorFrontalGyrus_male_70yrs_indiv53	superior frontal gyrus	male	70
normal	GSM300242	EntorhinalCortex_female_64yrs_indiv52	entorhinal cortex	female	64
normal	GSM300243	Hippocampus_female_64yrs_indiv52	hippocampus	female	64
normal	GSM300244	PostcentralGyrus_female_64yrs_indiv52	postcentral gyrus	female	64
normal	GSM300245	SuperiorFrontalGyrus_female_64yrs_indiv52	superior frontal gyrus	female	64
normal	GSM300238	EntorhinalCortex_female_70yrs_indiv47	entorhinal cortex	female	70
normal	GSM300239	Hippocampus_female_70yrs_indiv47	hippocampus	female	70
normal	GSM300240	PostcentralGyrus_female_70yrs_indiv47	postcentral gyrus	female	70
normal	GSM300241	SuperiorFrontalGyrus_female_70yrs_indiv47	superior frontal gyrus	female	70
normal	GSM300234	EntorhinalCortex_male_85yrs_indiv46	entorhinal cortex	male	85
normal	GSM300235	Hippocampus_male_85yrs_indiv46	hippocampus	male	85
normal	GSM300236	PostcentralGyrus_male_85yrs_indiv46	postcentral gyrus	male	85
normal	GSM300237	SuperiorFrontalGyrus_male_85yrs_indiv46	superior frontal gyrus	male	85
normal	GSM300230	EntorhinalCortex_female_37yrs_indiv45	entorhinal cortex	female	37
normal	GSM300231	Hippocampus_female_37yrs_indiv45	hippocampus	female	37
normal	GSM300232	PostcentralGyrus_female_37yrs_indiv45	postcentral gyrus	female	37
normal	GSM300233	SuperiorFrontalGyrus_female_37yrs_indiv45	superior frontal gyrus	female	37
normal	GSM300228	EntorhinalCortex_female_44yrs_indiv42	entorhinal cortex	female	44
normal	GSM300229	SuperiorFrontalGyrus_female_44yrs_indiv42	superior frontal gyrus	female	44
normal	GSM300227	Hippocampus_female_91yrs_indiv40-04	hippocampus	female	91
normal	GSM300226	PostcentralGyrus_female_90yrs_indiv40	postcentral gyrus	female	90
normal	GSM300222	EntorhinalCortex_female_74yrs_indiv38	entorhinal cortex	female	74
normal	GSM300223	Hippocampus_female_74yrs_indiv38	hippocampus	female	74
normal	GSM300224	PostcentralGyrus_female_74yrs_indiv38	postcentral gyrus	female	74

normal	GSM300225	SuperiorFrontalGyrus_female_74yrs_indiv38	superior frontal gyrus	female	74
normal	GSM300218	EntorhinalCortex_female_34yrs_indiv35	entorhinal cortex	female	34
normal	GSM300219	Hippocampus_female_34yrs_indiv35	hippocampus	female	34
normal	GSM300220	PostcentralGyrus_female_34yrs_indiv35	postcentral gyrus	female	34
normal	GSM300221	SuperiorFrontalGyrus_female_34yrs_indiv35	superior frontal gyrus	female	34
normal	GSM300214	EntorhinalCortex_male_97yrs_indiv34	entorhinal cortex	male	97
normal	GSM300215	Hippocampus_male_97yrs_indiv34	hippocampus	male	97
normal	GSM300216	PostcentralGyrus_male_97yrs_indiv34	postcentral gyrus	male	97
normal	GSM300217	SuperiorFrontalGyrus_male_97yrs_indiv34	superior frontal gyrus	male	97
normal	GSM300212	PostcentralGyrus_female_74yrs_indiv30	postcentral gyrus	female	74
normal	GSM300213	SuperiorFrontalGyrus_female_74yrs_indiv30	superior frontal gyrus	female	74
normal	GSM300208	EntorhinalCortex_male_28yrs_indiv29	entorhinal cortex	male	28
normal	GSM300209	Hippocampus_male_28yrs_indiv29	hippocampus	male	28
normal	GSM300210	PostcentralGyrus_male_28yrs_indiv29	postcentral gyrus	male	28
normal	GSM300211	SuperiorFrontalGyrus_male_28yrs_indiv29	superior frontal gyrus	male	28
normal	GSM300204	EntorhinalCortex_male_83yrs_indiv28	entorhinal cortex	male	83
normal	GSM300205	Hippocampus_male_83yrs_indiv28	hippocampus	male	83
normal	GSM300206	PostcentralGyrus_male_83yrs_indiv28	postcentral gyrus	male	83
normal	GSM300207	SuperiorFrontalGyrus_male_83yrs_indiv28	superior frontal gyrus	male	83
normal	GSM300202	PostcentralGyrus_female_91yrs_indiv27	postcentral gyrus	female	91
normal	GSM300203	SuperiorFrontalGyrus_female_91yrs_indiv27	superior frontal gyrus	female	91
normal	GSM300200	PostcentralGyrus_female_92yrs_indiv26	postcentral gyrus	female	92
normal	GSM300201	SuperiorFrontalGyrus_female_92yrs_indiv26	superior frontal gyrus	female	92
normal	GSM300196	EntorhinalCortex_female_74yrs_indiv21	entorhinal cortex	female	74
normal	GSM300197	Hippocampus_female_74yrs_indiv21	hippocampus	female	74
normal	GSM300198	PostcentralGyrus_female_74yrs_indiv21	postcentral gyrus	female	74
normal	GSM300199	SuperiorFrontalGyrus_female_74yrs_indiv21	superior frontal gyrus	female	74
normal	GSM300192	EntorhinalCortex_female_99yrs_indiv2	entorhinal cortex	female	99
normal	GSM300193	Hippocampus_female_99yrs_indiv2	hippocampus	female	99
normal	GSM300194	PostcentralGyrus_female_99yrs_indiv2	postcentral gyrus	female	99
normal	GSM300195	SuperiorFrontalGyrus_female_99yrs_indiv2	superior frontal gyrus	female	99
normal	GSM300189	EntorhinalCortex_female_74yrs_indiv18	entorhinal cortex	female	74
normal	GSM300190	Hippocampus_female_74yrs_indiv18	hippocampus	female	74
normal	GSM300191	SuperiorFrontalGyrus_female_74yrs_indiv18	superior frontal gyrus	female	74
normal	GSM300186	EntorhinalCortex_female_45yrs_indiv17	entorhinal cortex	female	45
normal	GSM300187	Hippocampus_female_45yrs_indiv17	hippocampus	female	45
normal	GSM300188	SuperiorFrontalGyrus_female_45yrs_indiv17	superior frontal gyrus	female	45
normal	GSM300185	Hippocampus_male_91yrs_indiv16-07	hippocampus	male	91
normal	GSM300181	EntorhinalCortex_male_80yrs_indiv15	entorhinal cortex	male	80
normal	GSM300182	Hippocampus_male_80yrs_indiv15	hippocampus	male	80

normal	GSM300183	PostcentralGyrus_male_80yrs_indiv15	postcentral gyrus	male	80
normal	GSM350078	SuperiorFrontalGyrus_male_80yrs_indiv15	superior frontal gyrus	male	80
normal	GSM300177	EntorhinalCortex_male_95yrs_indiv14	entorhinal cortex	male	95
normal	GSM300178	Hippocampus_male_95yrs_indiv14	hippocampus	male	95
normal	GSM300179	PostcentralGyrus_male_95yrs_indiv14	postcentral gyrus	male	95
normal	GSM300180	SuperiorFrontalGyrus_male_95yrs_indiv14	superior frontal gyrus	male	95
normal	GSM300173	EntorhinalCortex_male_45yrs_indiv12	entorhinal cortex	male	45
normal	GSM300174	Hippocampus_male_45yrs_indiv12	hippocampus	male	45
normal	GSM300175	PostcentralGyrus_male_45yrs_indiv12	postcentral gyrus	male	45
normal	GSM300176	SuperiorFrontalGyrus_male_45yrs_indiv12	superior frontal gyrus	male	45
normal	GSM300172	Hippocampus_female_82yrs_indiv111	hippocampus	female	82
normal	GSM300171	Hippocampus_female_87yrs_indiv109	hippocampus	female	87
normal	GSM300170	Hippocampus_male_84yrs_indiv108	hippocampus	male	84
normal	GSM300169	Hippocampus_male_82yrs_indiv106	hippocampus	male	82
normal	GSM300168	Hippocampus_female_96yrs_indiv105	hippocampus	female	96
normal	GSM300166	PostcentralGyrus_female_91yrs_indiv10	postcentral gyrus	female	91
normal	GSM300167	SuperiorFrontalGyrus_female_91yrs_indiv10	superior frontal gyrus	female	91
Alzheimer's Disease	GSM1176196	entorhinal cortex_female_76_AD_48	entorhinal cortex	female	76
Alzheimer's Disease	GSM1176197	entorhinal cortex_female_86_AD_15	entorhinal cortex	female	86
Alzheimer's Disease	GSM1176198	entorhinal cortex_female_82_AD_9	entorhinal cortex	female	82
Alzheimer's Disease	GSM1176199	entorhinal cortex_female_85_AD_24	entorhinal cortex	female	85
Alzheimer's Disease	GSM1176200	entorhinal cortex_female_90_AD_36	entorhinal cortex	female	90
Alzheimer's Disease	GSM1176201	entorhinal cortex_female_90_AD_6	entorhinal cortex	female	90
Alzheimer's Disease	GSM1176202	entorhinal cortex_female_90_AD_75	entorhinal cortex	female	90
Alzheimer's Disease	GSM1176203	entorhinal cortex_female_91_AD_11	entorhinal cortex	female	91
Alzheimer's Disease	GSM1176204	entorhinal cortex_male_76_AD_69	entorhinal cortex	male	76
Alzheimer's Disease	GSM1176205	entorhinal cortex_male_85_AD_51	entorhinal cortex	male	85
Alzheimer's Disease	GSM1176206	entorhinal cortex_male_85_AD_74	entorhinal cortex	male	85
Alzheimer's Disease	GSM1176207	entorhinal cortex_male_86_AD_92	entorhinal cortex	male	86
Alzheimer's Disease	GSM1176208	entorhinal cortex_male_87_AD_19	entorhinal cortex	male	87
Alzheimer's Disease	GSM1176209	entorhinal cortex_male_94_AD_5	entorhinal cortex	male	94
Alzheimer's Disease	GSM1176210	entorhinal cortex_male_94_AD_90	entorhinal cortex	male	94
Alzheimer's Disease	GSM1176211	hippocampus_female_60_AD_20	hippocampus	female	60
Alzheimer's Disease	GSM1176212	hippocampus_female_74_AD_37	hippocampus	female	74
Alzheimer's Disease	GSM1176213	hippocampus_female_76_AD_48	hippocampus	female	76
Alzheimer's Disease	GSM1176214	hippocampus_female_79_AD_16	hippocampus	female	79
Alzheimer's Disease	GSM1176215	hippocampus_female_86_AD_15	hippocampus	female	86
Alzheimer's Disease	GSM1176216	hippocampus_female_85_AD_24	hippocampus	female	85
Alzheimer's Disease	GSM1176217	hippocampus_female_90_AD_23	hippocampus	female	90
Alzheimer's Disease	GSM1176218	hippocampus_female_90_AD_36	hippocampus	female	90

Alzheimer's Disease	GSM1176219	hippocampus_female_90_AD_75	hippocampus	female	90
Alzheimer's Disease	GSM1176220	hippocampus_female_91_AD_11	hippocampus	female	91
Alzheimer's Disease	GSM1176221	hippocampus_male_76_AD_33	hippocampus	male	76
Alzheimer's Disease	GSM1176222	hippocampus_male_76_AD_69	hippocampus	male	76
Alzheimer's Disease	GSM1176223	hippocampus_male_79_AD_7	hippocampus	male	79
Alzheimer's Disease	GSM1176224	hippocampus_male_80_AD_43	hippocampus	male	80
Alzheimer's Disease	GSM1176225	hippocampus_male_85_AD_51	hippocampus	male	85
Alzheimer's Disease	GSM1176226	hippocampus_male_86_AD_92	hippocampus	male	86
Alzheimer's Disease	GSM1176227	hippocampus_male_87_AD_19	hippocampus	male	87
Alzheimer's Disease	GSM1176228	hippocampus_male_94_AD_5	hippocampus	male	94
Alzheimer's Disease	GSM1176229	hippocampus_male_94_AD_90	hippocampus	male	94
Alzheimer's Disease	GSM1176230	post-central gyrus_female_60_AD_20	post-central gyrus	female	60
Alzheimer's Disease	GSM1176231	post-central gyrus_female_74_AD_37	post-central gyrus	female	74
Alzheimer's Disease	GSM1176232	post-central gyrus_female_76_AD_48	post-central gyrus	female	76
Alzheimer's Disease	GSM1176233	post-central gyrus_female_86_AD_15	post-central gyrus	female	86
Alzheimer's Disease	GSM1176234	post-central gyrus_female_82_AD_13	post-central gyrus	female	82
Alzheimer's Disease	GSM1176235	post-central gyrus_female_82_AD_9	post-central gyrus	female	82
Alzheimer's Disease	GSM1176236	post-central gyrus_female_85_AD_24	post-central gyrus	female	85
Alzheimer's Disease	GSM1176237	post-central gyrus_female_85_AD_57	post-central gyrus	female	85
Alzheimer's Disease	GSM1176238	post-central gyrus_female_90_AD_23	post-central gyrus	female	90
Alzheimer's Disease	GSM1176239	post-central gyrus_female_90_AD_36	post-central gyrus	female	90
Alzheimer's Disease	GSM1176240	post-central gyrus_female_90_AD_6	post-central gyrus	female	90
Alzheimer's Disease	GSM1176241	post-central gyrus_female_90_AD_75	post-central gyrus	female	90
Alzheimer's Disease	GSM1176242	post-central gyrus_female_91_AD_11	post-central gyrus	female	91
Alzheimer's Disease	GSM1176243	post-central gyrus_female_94_AD_70	post-central gyrus	female	94
Alzheimer's Disease	GSM1176244	post-central gyrus_female_95_AD_64	post-central gyrus	female	95
Alzheimer's Disease	GSM1176245	post-central gyrus_male_76_AD_33	post-central gyrus	male	76
Alzheimer's Disease	GSM1176246	post-central gyrus_male_76_AD_69	post-central gyrus	male	76
Alzheimer's Disease	GSM1176247	post-central gyrus_male_79_AD_7	post-central gyrus	male	79
Alzheimer's Disease	GSM1176248	post-central gyrus_male_85_AD_51	post-central gyrus	male	85
Alzheimer's Disease	GSM1176249	post-central gyrus_male_85_AD_74	post-central gyrus	male	85
Alzheimer's Disease	GSM1176250	post-central gyrus_male_86_AD_92	post-central gyrus	male	86
Alzheimer's Disease	GSM1176251	post-central gyrus_male_87_AD_19	post-central gyrus	male	87
Alzheimer's Disease	GSM1176252	post-central gyrus_male_94_AD_44	post-central gyrus	male	94
Alzheimer's Disease	GSM1176253	post-central gyrus_male_94_AD_5	post-central gyrus	male	94
Alzheimer's Disease	GSM1176254	post-central gyrus_male_94_AD_90	post-central gyrus	male	94
Alzheimer's Disease	GSM1176255	superior frontal gyrus_female_74_AD_37	superior frontal gyrus	female	74
Alzheimer's Disease	GSM1176256	superior frontal gyrus_female_76_AD_48	superior frontal gyrus	female	76
Alzheimer's Disease	GSM1176257	superior frontal gyrus_female_86_AD_15	superior frontal gyrus	female	86
Alzheimer's Disease	GSM1176258	superior frontal gyrus_female_82_AD_9	superior frontal gyrus	female	82

Alzheimer's Disease	GSM1176259	superior frontal gyrus_female_85_AD_24	superior frontal gyrus	female	85
Alzheimer's Disease	GSM1176260	superior frontal gyrus_female_85_AD_57	superior frontal gyrus	female	85
Alzheimer's Disease	GSM1176261	superior frontal gyrus_female_88_AD_91	superior frontal gyrus	female	88
Alzheimer's Disease	GSM1176262	superior frontal gyrus_female_90_AD_23	superior frontal gyrus	female	90
Alzheimer's Disease	GSM1176263	superior frontal gyrus_female_90_AD_36	superior frontal gyrus	female	90
Alzheimer's Disease	GSM1176264	superior frontal gyrus_female_90_AD_6	superior frontal gyrus	female	90
Alzheimer's Disease	GSM1176265	superior frontal gyrus_female_90_AD_75	superior frontal gyrus	female	90
Alzheimer's Disease	GSM1176266	superior frontal gyrus_female_91_AD_11	superior frontal gyrus	female	91
Alzheimer's Disease	GSM1176267	superior frontal gyrus_female_94_AD_70	superior frontal gyrus	female	94
Alzheimer's Disease	GSM1176268	superior frontal gyrus_female_95_AD_64	superior frontal gyrus	female	95
Alzheimer's Disease	GSM1176269	superior frontal gyrus_male_76_AD_33	superior frontal gyrus	male	76
Alzheimer's Disease	GSM1176270	superior frontal gyrus_male_85_AD_51	superior frontal gyrus	male	85
Alzheimer's Disease	GSM1176271	superior frontal gyrus_male_85_AD_74	superior frontal gyrus	male	85
Alzheimer's Disease	GSM1176272	superior frontal gyrus_male_86_AD_92	superior frontal gyrus	male	86
Alzheimer's Disease	GSM1176273	superior frontal gyrus_male_94_AD_44	superior frontal gyrus	male	94
Alzheimer's Disease	GSM1176274	superior frontal gyrus_male_94_AD_5	superior frontal gyrus	male	94
Alzheimer's Disease	GSM1176275	superior frontal gyrus_male_94_AD_90	superior frontal gyrus	male	94
