# Supplementary material for: Integrated analysis and identification of hub genes as novel biomarkers for Alzheimer’s disease
Source: Front Aging Neurosci. 2022 Aug 30;14:901972. doi: 10.3389/fnagi.2022.901972 (PMC9468260; doi:10.3389/fnagi.2022.901972)
Supplement: Supplementary file 3 [file Data_Sheet_3.pdf]

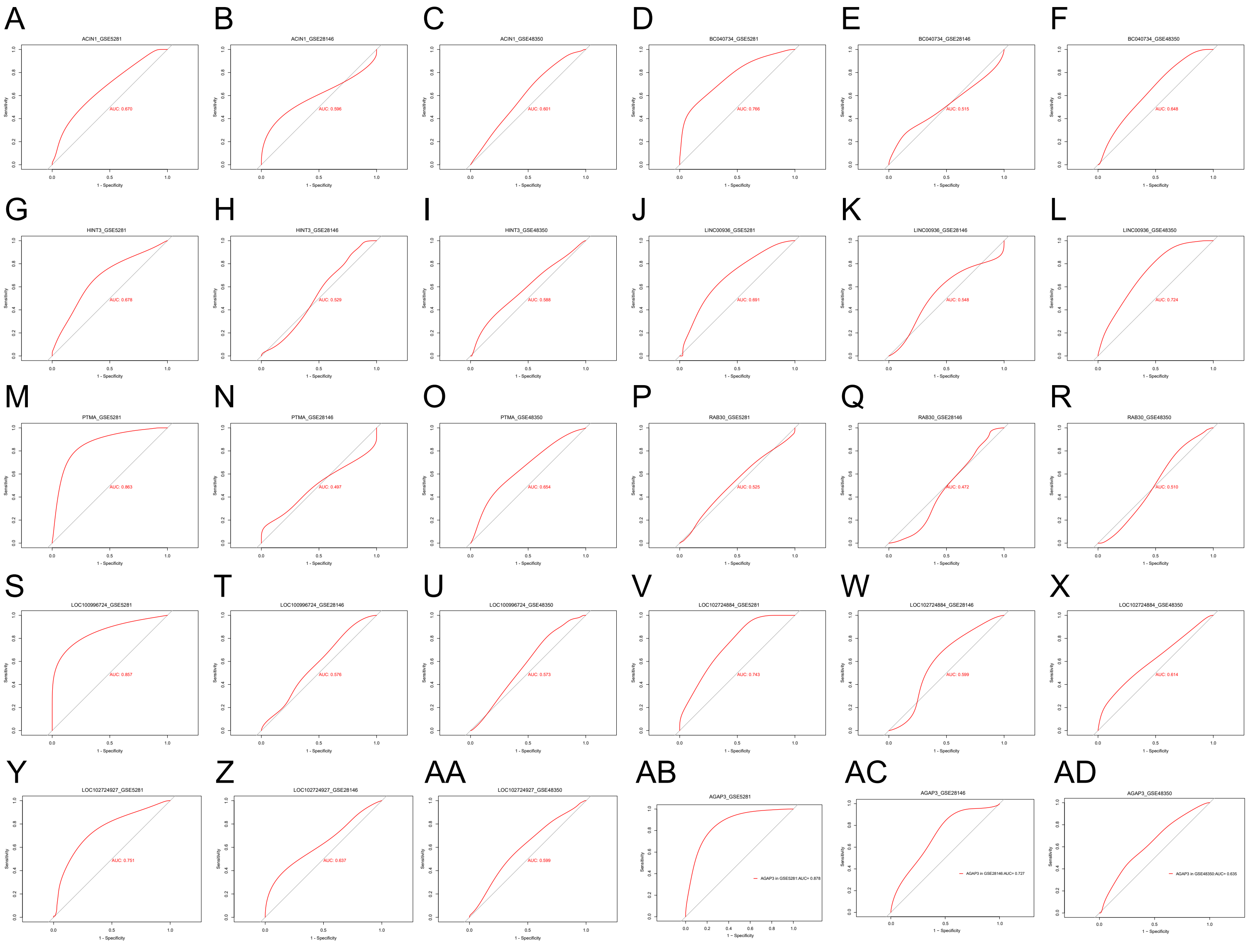

A
ACIN1_GSE5281
AUC: 0.670
B
ACIN1_GSE28146
AUC: 0.596
C
ACIN1_GSE48350
AUC: 0.601
D
BC040734_GSE5281
AUC: 0.766
E
BC040734_GSE28146
AUC: 0.515
F
BC040734_GSE48350
AUC: 0.648
G
HINT3_GSE5281
AUC: 0.678
H
HINT3_GSE28146
AUC: 0.529
I
HINT3_GSE48350
AUC: 0.588
J
LINC00936_GSE5281
AUC: 0.691
K
LINC00936_GSE28146
AUC: 0.548
L
LINC00936_GSE48350
AUC: 0.724
M
PTMA_GSE5281
AUC: 0.863
N
PTMA_GSE28146
AUC: 0.497
O
PTMA_GSE48350
AUC: 0.654
P
RAB30_GSE5281
AUC: 0.525
Q
RAB30_GSE28146
AUC: 0.472
R
RAB30_GSE48350
AUC: 0.510
S
LOC100996724_GSE5281
AUC: 0.857
T
LOC100996724_GSE28146
AUC: 0.576
U
LOC100996724_GSE48350
AUC: 0.573
V
LOC102724884_GSE5281
AUC: 0.743
W
LOC102724884_GSE28146
AUC: 0.599
X
LOC102724884_GSE48350
AUC: 0.614
Y
LOC102724927_GSE5281
AUC: 0.751
Z
LOC102724927_GSE28146
AUC: 0.637
AA
LOC102724927_GSE48350
AUC: 0.599
AB
AGAP3_GSE5281
AGAP3 in GSE5281:AUC= 0.878
AC
AGAP3_GSE28146
AGAP3 in GSE28146:AUC= 0.727
AD
AGAP3_GSE48350
AGAP3 in GSE48350:AUC= 0.635
Sensitivity
1 - Specificity

Figure S1. The ROC curves of 10 genes in the three datasets. **(A)**. The ROC curve of ACIN1 in GSE5281. **(B)**. The ROC curve of ACIN1 in GSE28146. **(C)**. The ROC curve of ACIN1 in GSE48350. **(D)**. The ROC curve of BC040734 in GSE5281. **(E)**. The ROC curve of BC040734 in GSE28146. **(F)**. The ROC curve of BC040734 in GSE48350. **(G)**. The ROC curve of HINT3 in GSE5281. **(H)**. The ROC curve of HINT3 in GSE28146. **(I)**. The ROC curve of HINT3 in GSE48350. **(J)**. The ROC curve of LINC00936 in GSE5281. **(K)**. The ROC curve of LINC00936 in GSE28146. **(L)**. The ROC curve of LINC00936 in GSE48350. **(M)**. The ROC curve of PTMA in GSE5281. **(N)**. The ROC curve of PTMA in GSE28146. **(O)**. The ROC curve of PTMA in GSE48350. **(P)**. The ROC curve of RAB30 in GSE5281. **(Q)**. The ROC curve of RAB30 in GSE28146. **(R)**. The ROC curve of RAB30 in GSE48350. **(S)**. The ROC curve of LOC100996724 in GSE5281. **(T)**. The ROC curve of LOC100996724 in GSE28146. **(U)**. The ROC curve of LOC100996724 in GSE48350. **(V)**. The ROC curve of LOC102724884 in GSE5281. **(W)**. The ROC curve of LOC102724884 in GSE28146. **(X)**. The ROC curve of LOC102724884 in GSE48350. **(Y)**. The ROC curve of LOC102724927 in GSE5281. **(Z)**. The ROC curve of LOC102724927 in GSE28146. **(AA)**. The ROC curve of LOC102724927 in GSE48350. **(AB)**. The ROC curve of AGAP3 in GSE5281. **(AC)**. The ROC curve of AGAP3 in GSE28146. **(AD)**. The ROC curve of AGAP3 in GSE48350.
